# Subsurface *In Situ* Detection of Microbes and Diverse Organic Matter Hotspots in the Greenland Ice Sheet

**DOI:** 10.1089/ast.2020.2241

**Published:** 2020-10-09

**Authors:** Michael J. Malaska, Rohit Bhartia, Kenneth S. Manatt, John C. Priscu, William J. Abbey, Boleslaw Mellerowicz, Joseph Palmowski, Gale L. Paulsen, Kris Zacny, Evan J. Eshelman, Juliana D'Andrilli

**Affiliations:** ^1^ Jet Propulsion Laboratory/California Institute of Technology, Pasadena, California, USA.; ^2^ Photon Systems, Inc., Covina, California, USA.; ^3^ Department of Land Resources & Environmental Sciences, Montana State University, Bozeman, Montana, USA.; ^4^ Honeybee Robotics, Altadena, California, USA.; ^5^ Impossible Sensing, St. Louis, Missouri, USA.; ^6^ Louisiana Universities Marine Consortium, Chauvin, Louisiana, USA.

**Keywords:** Deep-UV spectroscopy, Fluorescence mapping, Organic detection, Europa, Enceladus, Titan. Astrobiology 20, 1185–1211

## Abstract

We used a deep-ultraviolet fluorescence mapping spectrometer, coupled to a drill system, to scan from the surface to 105 m depth into the Greenland ice sheet. The scan included firn and glacial ice and demonstrated that the instrument is able to determine small (mm) and large (cm) scale regions of organic matter concentration and discriminate spectral types of organic matter at high resolution. Both a linear point cloud scanning mode and a raster mapping mode were used to detect and localize microbial and organic matter “hotspots” embedded in the ice. Our instrument revealed diverse spectral signatures. Most hotspots were <20 mm in diameter, clearly isolated from other hotspots, and distributed stochastically; there was no evidence of layering in the ice at the fine scales examined (100 μm per pixel). The spectral signatures were consistent with organic matter fluorescence from microbes, lignins, fused-ring aromatic molecules, including polycyclic aromatic hydrocarbons, and biologically derived materials such as fulvic acids. *In situ* detection of organic matter hotspots in ice prevents loss of spatial information and signal dilution when compared with traditional bulk analysis of ice core meltwaters. Our methodology could be useful for detecting microbial and organic hotspots in terrestrial icy environments and on future missions to the Ocean Worlds of our Solar System.

## 1. Introduction

Ice is an important environment for life on Earth and possibly elsewhere in the Solar System (Priscu and Hand, 2012; Garcia-Lopez and Cid, 2017). Studies of terrestrial glacial ice have revealed microenvironments containing viable microbes that are both preserved and actively metabolizing (Skidmore *et al.*, 2000; Campen *et al.*, 2003; Miteva and Brenchley, 2005; Miteva, 2008; Liu *et al.*, 2016, 2018). These microhabitats exist in a wide variety of icy environments: snow, glacial ice, deep glacial ice, frozen ponds, sea ice, etc. (Margesin and Miteva, 2011; Margesin and Collins, 2019). For example, glacial ice cores collected from the West Antarctic Ice Sheet (WAIS) Divide borehole in Antarctica contained prokaryotic cell concentrations from 10^4^ to 10^6^ cells/cm^3^ at a depth of 1.7–2.7 km below the surface (Santibáñez *et al.*, 2018). Similarly, glacial ice from the Greenland Ice Sheet Project 2 (GISP2) borehole had cellular concentrations of 10^4^–10^6^ cells/cm^3^ from 1.5 to 2.5 km below the surface (Miteva *et al.*, 2009). Priscu and Christner (2004) and Priscu *et al.* (2009) used estimates of glacier ice cellular concentrations and ice volume to show that the Antarctica and Greenland ice sheets combined represent nearly 4.4 × 10^24^ prokaryotic cells, which equates to 4.8 × 10^−5^ Pg of organic carbon—nearly the equivalent carbon biomass of all the liquid freshwater lakes on Earth (Whitman *et al.*, 1998; Priscu and Christner, 2004). Much of the habitable space inside ice sheets is found in liquid channels and junctions between ice grains; Barletta *et al.* (2012) estimated that intergrain liquid water channels in the Antarctic and Greenland ice sheet represent 16.7 and 576 km^3^, respectively, of habitable space that can support microbial populations.

With temperature and pressure conditions similar to the deep interiors of ice sheets on Earth, the deep convecting icy crusts of the Ocean Worlds of the Solar System (Europa, Enceladus, Titan) may also be habitable environments. The deep ice of the Outer Solar system satellites would be the first potentially habitable environment encountered by future missions targeting the deep subsurface oceans of those worlds (Marion *et al.*, 2003; Priscu and Hand, 2012; Vance *et al.*, 2018). Exploring the various icy habitats in terrestrial subsurface ice will lead to a better understanding of the potential for life in the icy environments on Earth and also constrain our understanding of how microbes and organic biomolecules can be preserved in the icy regions of planetary bodies (*e.g*., Mars' poles, Europa, Enceladus, Titan, etc.) (Priscu *et al.*, 1998, 1999; Price, 2006; Priscu and Hand, 2012; Boetius *et al.*, 2015; D'Andrilli *et al.*, 2017; Garcia-Lopez and Cid, 2017). In particular, understanding how organic materials (nutrients, building blocks) and microbial cells in terrestrial ice sheets are distributed and interrelated in ice microenvironments (Priscu *et al.*, 2007; Margesin and Collins, 2019) will aid in the exploration of these alien worlds.

The Greenland ice sheet consists of meteorically derived ice that can serve as an analogue for other icy locations in the Solar System. The high altitude (3211 m) and low temperatures (−48°C average winter minimum to −11°C average summer maximum) at the top of the ice sheet represent a textbook example of dry (minimal liquid) deposition, compression, and modification of grains to form ice (see Lomonaco *et al.*, 2011; Baker, 2019). At the surface, the ice is porous and consists of a series of interlocked grains known as firn, whereas deeper, the grains become altered as the pore spaces close off and firn converts to nonporous glacial ice.

Previous work by Lomonaco *et al.* (2011) shows that air pockets seal off completely from exchange with the surface at a depth of 69 m (referred to as the lock-in zone) whereas the pore close-off occurs at 81 m. These same processes can occur on Mars where deposited ice material accumulates and compresses. They may also occur on Saturn's moon Enceladus (and possibly Jupiter's moon Europa) in areas near jets where ballistically ejected water ice accumulates back onto the surface. It is possible that the entire history of ejected organic and other materials of Enceladus' jets could be recorded in these near-jet re-deposition terrains, just like the snowfall record on Earth. Although the lower gravity and lower temperatures compared with Earth will affect the corresponding depth where porous firn transitions to nonporous ice, the same fundamental physical and chemical principles will apply (see Vance *et al.*, 2018 for predicted T and P curves for the Ocean Worlds in the outer Solar System). Deposition and compression, but minimal lateral movement and fluid percolation, make the top of the Greenland ice sheet an ideal location to study the processes involved in the transition of snow to firn to glacial ice, and the materials trapped within (Meese *et al.*, 1994; McGrath *et al.*, 2013).

Research on natural and laboratory terrestrial ice has shown that microbes in ice are found localized in brine channels and triple junctions between ice grains (Price, 2000; Junge *et al.*, 2001; Mader *et al.*, 2006; Barletta and Roe, 2012; Barletta *et al.*, 2012). For water-soluble materials such as salts, as water is incorporated into growing ice grains, soluble materials are excluded and pushed into a network of micron-scale inter-grain channels that surround the ice grains (Price, 2000; Barletta and Roe, 2012; Barletta *et al.*, 2012; Santibáñez *et al.*, 2019). Barletta *et al.* (2012) used micro-Raman spectroscopy to show that sulfate and nitrate concentrations in the channels of glacial ice from the GISP2 and Antarctic alpine glaciers were between 10^4^ and 10^5^ times greater than the concentrations measured in the bulk meltwater.

For insoluble materials, laboratory experiments show that the size of solid grains plays an important role, with grains <5 μm becoming partitioned into the brine channels, and grains >5 μm becoming embedded in the growing ice crystal (Mader *et al.*, 2006). However, recent work by Santibáñez *et al.* (2019) demonstrated that microbes, both alive and dead, along with more simple organic materials, will be incorporated into lake ice during freezing, suggesting that more complex partitioning processes may occur. Work by Pinto *et al.* (2019) showed that local concentrations of organic molecules can influence the types of microbes present in icy environments such as snow where organic emplacement determines the makeup of the microbial community. Thus, understanding organic partitioning in ice can provide key information about the ice microenvironment that determines the microbial population.

Each of these observations and experiments show that ice formation and subsequent modification have the ability to partition soluble and insoluble materials and microbes. For example, partitioning coefficients between ice and water during the freezing process have been used to predict the geochemistry and bacterial density in Vostok Subglacial Lake, which lies beneath ∼4 km of ice in East Antarctica (Priscu *et al.*, 1999). However, what is not known is the ultimate partitioning and distribution of microbes and organic materials in glacial ice (*i.e*. *in situ*) at fine spatial scales in microhabitats (Margesin and Collins, 2019). It is likely that the first samples for the ocean worlds in the outer Solar System will be obtained from their icy shells. Hence, it is imperative that methods be developed and deployed to these frozen worlds that can characterize organic matter within the ice, which then can be used together with a knowledge of crustal dynamics to predict conditions in the underlying deep subsurface ocean.

Key questions we attempted to answer in our present study include: Are the naturally fluorescent microbes and other organics within the ice confined to discrete layers due to initial deposition? Are higher concentrations also associated with bubbles or emplaced in other discrete spots? Does the transformation from firn to glacial ice alter the distribution of fluorescent materials? Is the distribution of fluorescent materials the same in firn ice as it is in glacial ice?

In the past, these questions were difficult to answer. Traditionally, chemical and microbial analysis of ice cores is accomplished by extracting the ice core, then melting the ice core by continuous melting or melting of core sections, followed by chemical analysis of the melted sample at varying resolution (Christner *et al.*, 2005; Miteva and Brenchley, 2005; Tung *et al.*, 2005; Yung *et al.*, 2007; Miteva *et al.*, 2009; D'Andrilli *et al.*, 2017; Santibáñez *et al.*, 2018). Unfortunately, this procedure results in loss of high spatial resolution, dilution of analytes (often to undetectable levels) that are concentrated in microenvironments, and loss of critical information for discerning the mechanisms of emplacement and distribution. Any details at scales finer than that used to create meltwater that is sufficient for analysis are lost. Such information is critical to understanding the temporal sequence of paleoclimate in meteoric ice and for predicting conditions in underlying water bodies such as subglacial lakes on Earth and the ocean worlds of our Solar System. In addition, during core extraction and sample handling there is the risk of physical damage from melting, cracking from pressure release, and contamination from extraction, bagging, and transport (Tung *et al.*, 2005). Decontamination procedures exist for ice cores, but they require removing ∼2 cm of the outer ice core, resulting in significant sample loss (Christner *et al.*, 2005). For exploration of planetary surfaces beyond Earth, extraction of ice cores and shipment back to a laboratory is complex and likely impossible; thus, an *in situ* measurement technique is desirable.

The overarching objective of our work is to determine the fine-scale distribution of cellular and noncellular organic matter without ice core extraction, transport, melting, and the associated risks of contamination. To address this objective, we developed a deep-ultraviolet (DUV) fluorescence Raman mapping spectrometer for *in situ* analysis (Eshelman *et al.*, 2019). Spatially resolved DUV fluorescence spectroscopy of ice is a powerful tool for the identification and localization of different molecular species based on electronic transitions (Rohde and Price, 2007; Bhartia *et al.*, 2008, 2010; Rohde *et al.*, 2008; Price, 2009; Rohde, 2010; Barletta and Roe 2012; Barletta *et al.*, 2012; Eshelman *et al.*, 2019). For *in situ* exploration, we coupled a DUV fluorescence mapping spectrometer with a wireline robotic drill system to create a down-borehole instrument-drill combination referred to as WATSON (Wireline Analysis Tool for the Subsurface Observation of Northern ice sheets). This combination is a departure from traditional ice exploration where a sample is acquired and delivered to an instrument: WATSON brings an instrument to an *in situ* sample.

In July of 2019, we used WATSON to drill a borehole and make fine-scale *in situ* measurements of the borehole wall to determine the distribution of fluorescent organic materials in firn and glacial ice to a depth of 107 m near Summit Station, Greenland. Our investigations at Summit Station provide new information on the processes that affect localization (and concentration) of organic and putative biosignatures during meteoric deposition and compaction. In addition, our efforts advanced detection and characterization techniques that could be used in a laboratory setting to identify hotspots in extracted ice cores for further analysis.

## 2. Methods

### 2.1. Summit Station, Greenland

Summit Station was established in 1989 as part of the GISP2 drill site. It is located near the apex of the Greenland ice sheet, just inside the southwestern boundary of the North East Greenland National Park (72.6°N, 38.5°W, elevation 3211 m [10,535′] EGM96, August 2019) (S. Dorsi, personal communication; Summit Guide, 2019). The Station is currently maintained by the U.S.-based CH2M HILL Polar Services, with support from the National Science Foundation, for the purpose of providing year-round support for scientific research into glaciology, meteorology, atmospheric chemistry, and a wide range of other fields requiring long-term environmental observations. The climate at Summit is currently classified as “ice cap,” with no month during the year having a mean monthly temperature exceeding 0°C, with exceptions in 2019 (after the completion of our expedition), 2012, 1889, and 7 other times between 1250 and 750 AD (Meese *et al.*, 1994; McGrath *et al.*, 2013). During the course of our fieldwork (June 4 to July 10, 2019), the temperature at Summit Station averaged −10.5°C (13.1°F), with a minimum of −26.9°C (−16.4°F) and a maximum daily temperature of 0.9°C (33.6°F). Humidity during this same period averaged 82%, with a minimum of 55% and a maximum of 98%.

### 2.2. Drill site location

The WATSON team elected to begin its drill efforts in a pre-existing borehole to maximize the time spent interrogating deeper glacial ice, as opposed to drilling in a new location through firn. Our team drilled and extended the “Baker Borehole,” a borehole located 6.75 km from Summit Station at N72.59781, W38.62563 (August 2018). During a project led by Ian Baker (Dartmouth College), the borehole was drilled in 2017 with a UWM-IDDO Eclipse-Badger drill. It was “dry drilled”—meaning that no drilling fluid was used to prevent lithostatic collapse of the borehole. Baker (2019) acquired firn cores to a depth of ∼85 m to study firn densification mechanisms and microstructural evolution (Ian Baker, personal communication; see also Lomonaco *et al.*, 2011). At the end of their campaign, the borehole was capped with a cover to prevent snow accumulation in the borehole. In June 2019, the WATSON drill team extended the Baker Borehole from 84.5 to 110.5 m with the Planetary Deep Drill prototype (Zacny *et al.*, 2016; Mellerowicz *et al.*, 2018). On arrival at the drill site, our initial inspection revealed that the original hole had maintained its integrity and depth—therefore, no borehole reaming was required. The diameter of the extended borehole was 11.18 cm (4.4″). Down-borehole camera images (not part of WATSON instrument) detected a melt lens at 3.28 m depth, which we interpreted to be due to the 2012 surface melt (McGrath *et al.*, 2013).

### 2.3. WATSON instrument and drill combination

**FIG. 1:**
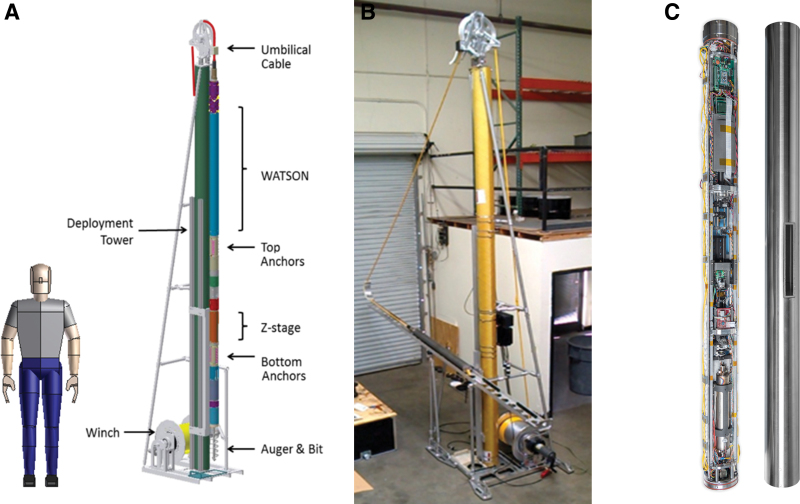
**(A–C)** Views of the instrument drill combination. **(A)** Schematic drawing of the Honeybee Planetary Deep Drill and WATSON DUV mapping spectrometer. Mannequin for scale. **(B)** Image of actual constructed drill and stand. **(C)** Image of WATSON DUV mapping spectrometer. Left side of image shows uncovered components, and right image shows instrument in down-borehole configuration with the protective tube in place. The optical window can be seen at center right. DUV, deep-ultraviolet; WATSON, Wireline Analysis Tool for the Subsurface Observation of Northern ice sheets. Color images are available online.

**FIG. 2.**
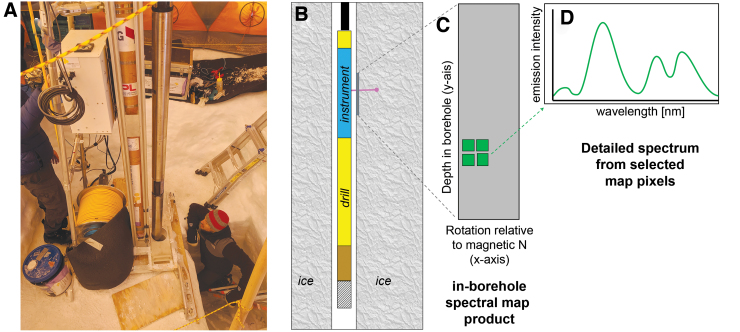
Image and schematic of operations showing in-borehole operation and data flow. **(A)** Image of WATSON drill instrument in drill tent at Summit Station, Greenland. The upright silver tube at the center is the WATSON instrument. Optical window at lower center in image. The rest of the drill is inside the borehole at lower center in image. **(B)** Cutaway schematic showing drill-instrument combination in the ice borehole. The WATSON instrument is indicated by a blue rectangle in the schematic. The laser illumination (not to scale) is indicated as a purple beam penetrating into ice and hitting a target. During a point cloud measurement, the instrument is moved vertically as the laser is illuminated along the length of the borehole. During a map, the instrument is stationary, and the laser beam is moved by a series of internal windows to build up a raster map product. **(C)** Graphic of a map product showing a portion of the borehole. Green squares indicate pixels of interest. **(D)** Cartoon simulating a spectral response extracted from one of the map pixels. Color images are available online.

WATSON consists of a DUV fluorescence Raman instrument coupled to the Honeybee Robotics Planetary Deep drill (Fig. 1). The 4 m long, 10 cm in diameter drill system combines a coring bit, power head, retractable anchor feet, and retractable skids at the lower end of a drill string, coupled to a DUV fluorescence Raman instrument with a mechanical stage for the optical mirror, as described in the work of Eshelman *et al.* (2019), at the upper end. For deployment (Fig. 2), WATSON is suspended on a power/communication tether from a drill platform and lowered down the borehole from the surface. An encoder attached to the tether measures the distance traveled and an onboard inertial measuring unit determines the rotation (thus orientation) inside the borehole relative to magnetic north. The communication tether allows commanding and data transfer with the surface. The drill system includes onboard electronics, sensors, and computers to monitor the drilling operation.

Before initial insertion into the ice borehole, the entire drill and spectrometer was thoroughly cleaned with 60–70% ethanol in water and wiped dry. The drilling operation starts with lowering the drill to the bottom of the borehole. Once at the bottom, the coring bit cuts a 50 cm-long ice core in approximately 1–2 minutes. The drill is then lifted off the hole bottom and during this step, core dogs cut and liberate the ice core from the substrate. The drill is subsequently lifted out of the hole where the core is removed, labeled, and stored for further analysis. The up and down operation is performed sequentially until the required depth is reached. During our drilling campaign, the average drilling rate was 6.5 m/day. We collected a total of 26 m of ice core, while exposing 26 m of fresh borehole wall for the WATSON instrument to interrogate. The average density of ice cores collected for this project, from 84.5 to 110.5 m, was 0.84 g/cm^3^; this is consistent with densities of glacial ice reported in the work of Bender *et al.* (1997), which have density values of 0.81–0.84 g/cm^3^ and air volumes of only 10–15%.

During instrument operation, a pulsed (40 μs) DUV (excitation wavelength: 248.6 nm) hollow-cathode NeCu laser was fired at 40 Hz through a series of optics that cleaned and shaped the laser beam and focused it through a fused silica optical window onto the borehole wall. This DUV illumination causes aromatic-containing organics to fluoresce at electronic transitions diagnostic of the electronic states of the particular molecule or mixture of molecules (Bhartia *et al.*, 2008; Rohde, 2010; Eshelman *et al.*, 2019). The fluorescence emission signal is collected by the same illuminating objective lens and reflected into a spectrometer coupled to a photomultiplier tube (PMT) array gated to the duration of the laser pulse. The spectral range observed is 275–450 nm with 32 equal bins. The instrument has an internal stage that allows raster scanning through an optical window. During scanning, operations involved collecting a dark (no laser) and an active acquisition during the 40 μs laser shot. This allowed subtraction of a dark background at a time close to the laser shot. Data collected included fluorescence intensity from each wavelength bin, depth, rotation, and a timestamp, as well as a flag for whether the signal was collected during a laser shot or as a dark. During point cloud operation, we favored slowly lowering the drill with skids in place to ensure rotational stability; however, there were times we acquired point clouds at a lower resolution where we allowed the drill to freely rotate inside the borehole. During detailed raster scanning, skids were engaged for rotational stability, and tether playout was prevented by using a brake to maintain a constant depth (Fig. 2).

### 2.4. Point cloud data acquisition

Point clouds were acquired as a series of successive laser shots as the drill-instrument system was lowered or raised at a constant rate with no movement of the internal mechanical stage. The physical spacing of shots varied between point clouds acquisitions as the laser firing rate and drill movement rate allowed varying spacing depending on desired resolution. We used sparse point shots with a post spacing varying between 5 mm for rapid surveys and 100 μm for more detailed spatial mapping. The points were thus distributed along a nearly vertical linear track during drill descent or ascent. During signal acquisition, the PMT voltage potential was kept constant at 800 mV.

We acquired 32 point clouds in the borehole covering the length of the borehole from 3.3 to 105.6 m depth. We targeted borehole depth coverage rather than rotational coverage. There were five gaps in depth coverage: from 0 to 3.3, 8.96 to 9.75, 10.48 to 19.81, 88.16 to 88.56, and 99.66 m to 99.78 m. As stated earlier, point clouds interleaved dark and active spectra. Since the entire drill-instrument combination, but not instrument mechanical stage, was in motion, the laser fired through the same section of the window during the scan. However, there was a wavelength-dependent background signal that needed to be removed from the acquired instrument signal intensity. This background also decreased during the course of a particular cloud or line scan run. During post-processing, we created a synthetic background for each point by determining average spectra of 10 successive laser shot reads after ∼500 laser shots and at the end of the run; the standard deviation of the 10 shots at each location was also recorded. After 500 shots, the background drift was in a linear regime. For each point, a synthetic background was created based on a linear interpolation of the average values at the two regions in the point cloud data, and this background was subtracted from the non-bias corrected linear spectra for each laser shot. (Bias offsets are already present in the uncorrected background.) The background generally had increased signals at both 324.6 and 341.2 nm and was never larger than 2400 counts in any of the recorded times of any of the point clouds. Supplementary Data S1 contains the full background-corrected point cloud dataset.

### 2.5. Map data acquisition and processing

Maps were acquired by locking WATSON at a constant depth inside the borehole by using metal skids and a serpentine raster line scan of the optical path across a target area. The scanning motor and laser repetition rate were synched such that the maps were prepared with a spatial scale of 100 μm per pixel. Typical map dimensions were 1 cm horizontal × 4 cm vertical and 3 cm horizontal × 4 cm vertical. As for the point cloud, raster maps signal acquisition interleaved dark and active spectra. After data collection, the laser-illuminated instrument counts were subtracted from the dark bias instrument counts and a multiband raster map was created.

During map analysis, the background spectra was removed for each identified spatial region of interest (ROI) in the mapped region. To do this, we selected two multi-pixel blank areas for each feature. These were approximately equidistant and were above and below the feature, usually within 5 pixels (0.5 mm) distance. The blank pixel values for each area were averaged, and the two sets were interpolated (averaged since equidistant to the target feature) to create a synthetic predicted background at the location of the identified feature. The synthetic predicted background was subtracted from the instrument response to determine the actual spectral response. The maximum standard deviation between the two blank areas was set as the noise level. During ROI determination, only those pixels above 3 × noise level for at least one of the bands were used to define the feature outline. For detailed spectral characterization, only those pixels that were 10 × noise level for the lambda max (λ_max_) and the main background peaks (either 324.6 or 341.2 nm) were selected for display. Supplementary Data S5 contains the complete set of points from the ROIs.

Later spatial calibration using a resolution target determined that acquired maps were compressed in the *z*-direction (*y*-direction in the *xy* maps of Fig. 9). Therefore, the dimensions in this direction were only 0.7 × the actual value. We compensated for this during angle and aspect ratio measurements by importing the uncompressed image into Photoshop (Adobe), spatially stretching the image in the *y*-direction, and recording the key dimensions and values with Photoshop tools. The map presented in Fig. 9 and the dataset in Supplementary Data S5 are spatially uncorrected in the depth direction—they represent the actual pixel coordinates directly obtained from the WATSON instrument.

**Table 4.** Hit Rate Calculated from Analysis of Map 1

Spectral type	Number regions	Percent of total hotspots	Areal coverage (pixels)	Areal % of total hotspots	Sum signal at lambda max (counts)	Percent of total signal	Hit rate frequency per 1000
L325_t	4	21.1	101	13.7	262,336	34.0	2.329
L325_s	1	5.3	38	5.2	18,668	2.4	0.876
L341_s	2	10.5	35	4.8	58,487	7.6	0.807
L374_sharp	1	5.3	9	1.2	17,247	2.2	0.208
L374_m	1	5.3	16	2.2	9099	1.2	0.369
L385m^a^	10	52.6	536	72.9	406,504	52.6	12.360

^a^ Features 4–6 and 14–16 are not combined in this analysis.

### 2.6. Fluorescence measurement of laboratory comparison standards

For comparison spectra, laboratory-acquired Excitation-Emission Matrices (EEMs) were obtained by using either a Hitachi F-4500 spectrometer or a Horiba Jobin-Yvon Fluoromax-4 Spectrofluorometer, equipped with an Xe lamp light source and a pathlength of 1 cm. The Hitachi F-4500 had an excitation wavelength scanning from 200 to 400 nm in 5 nm increments and intensity measurements from 200 to 600 nm every 2 nm with a scan speed of 1200 nm/min. Samples scanned by the Hitachi instrument included pure chemical standards as well as whole bacterial cultures. For pure chemical standards, homogeneous aqueous solutions were made and scanned; whereas for whole bacterial samples, culture suspensions in deionized water were used. Samples run on the Hitachi instrument included naphthalene, tryptophan, phenanthrene, anthracene, pyrene, methyl-1-pyrene, fluoranthene, perylene, *Shewanella oneidensis* MRI whole cells, and *Bacillus pumillus* whole cells.

The Horiba Jobin-Yvon Fluoromax-4 instrument used excitation wavelengths from 240 to 450 nm scanned over 10 nm intervals, with emission responses measured from 300 to 560 nm recorded in 2 nm increments. Samples run on the Horiba instrument included vanillic acid, *p*-benzoquinone, Suwannee River and Pony Lake fulvic acid samples, and an “Antarctic bacterium” isolate. Samples of the chemical solutions were prepared to a 20 mg/L stock solution, shaken for 24 h, and placed into a quartz cuvette; spectra were collected at 25°C. The “Antarctic bacterium” isolate is an extract from a sample collected from a supraglacial stream on the Cotton Glacier, Antarctica, and is described in the work of Dieser *et al.* (2019). The two fulvic acid reference samples were prepared as 2 mg/L solutions in ultrapure Milli-Q water. The Pony Lake fulvic acid reference sample is an Antarctic microbial organic matter end-member, originating from Pony Lake, Antarctica, and was obtained from the International Humic Substances Society; the Suwannee River fulvic acid standard is a continental organic matter end-member (Okefenokee Swamp), and it was also obtained from the International Humic Substances Society. Corresponding UV-absorbance spectra for these samples were also collected for EEMs post-processing analysis in accordance with protocols in the works of D'Andrilli *et al.* (2017) and Bhartia *et al.* (2008).

For compatibility, we extracted only the data coming from the 250 nm excitation wavelength from the three-dimensional fluorometer instruments (closest to the 248 nm excitation used in the WATSON drill-instrument combination). The recorded spectral emission responses were binned to match the WATSON wavelength binning and average values within the bin used. The spectra were then normalized to the highest response in the WATSON spectral range (275–425 nm), and this was used for comparison of WATSON spectra.

## 3. Results

### 3.1. Point cloud data analysis

**FIG. 3.**
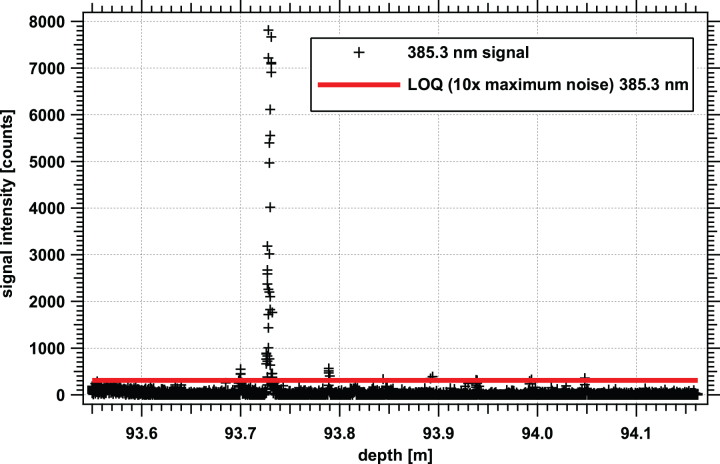
Example of background-subtracted point cloud data from WATSON. This plot shows the 385.3 nm data for Point Cloud 17 and was acquired at a depth from 93.5 to 94.2 m below the surface and with an average post-spacing of 153 μm. Data for photomultiplier tube responses for 4000 laser shots are represented here. For clarity, only data for the 385.3 nm band are shown. The red line indicates the value for 10 × the maximum standard deviation from the regions used for background determination. At this wavelength band, potential points of interest cluster at 93.70, 93.73, and 93.79 m. Color images are available online.

We identified points of interest in the point cloud data by searching for a clear discernable pattern when subtracted from the background spectrum. In general, this meant that at least three consecutive band responses were at least 10 times the maximal standard deviation from the two regions used for background determination. An example plot for the 385.3 nm wavelength band of the Point Cloud 17 dataset is shown in Fig. 3. Points above the 10 × noise level (above the red line in the figure) were deemed potential points of interest, and signals from neighboring bands were examined further.

Table 1 presents the summarized data for all 32 point clouds, including the hit rate for each 4000 laser shot cloud reported as the number of points (laser shot returns) of interest per 1000 laser shots. The hit rate value for each point cloud varies from a maximum of 73–0. The combined point cloud dataset represents the instrument response from 127,999 laser shots. Of these shots, only 1039 points were determined to be of interest for an overall ratio of targets of interest to shots fired of 0.8%. Supplementary Data S2 contains the spectral data for all the points of interest identified from the point clouds.

**Table 1.** Table of Point Cloud Datasets Collected Sorted by Depth

Point cloud map	Start depth (m)	Final depth (m)	Start rotational orientation (degrees from magnetic N)	Final rotational orientation (degrees from magnetic N)	Average post-spacing (μm)	No. of identified features	Hit rate per thousand laser shots^a^
1	8.964	3.353^b^	34	355	−1403^b^	42	10.5
2	9.752	10.482	68	62	183	64	16
3	19.811	20.295	78	69	121	37	9.25
4	20.002	26.297	61	57	1574	11	2.75
5	26.41	47.687	44	66	5321	106	26.5
6	29.144	35.287	130	173	1536	40	10
7	39.819	46.352	154	148	1634	10	2.5
8	48.207	68.368	51	75	5042	10	2.5
9	55.219	61.395	67	91	1544	20	5
10	65.338	72.331	3	186	1749	6	1.5
11	68.913	88.162	66	85	4813	25	6.25 (6)^c^
12	76.966	86.727	323	350	2441	67	16.75
13	83.75	84.183	35	35	108	0	0
14	84.549	85.048	34	34	125	292	73 (13.5)^c^
15	88.566	94.043	73	67	1370	36	9
16	89.225	93.879	4	62	1164	19	4.75
17	93.549	94.161	48	48	153	50	12.5
18	93.549	94.217	33	33	167	4	1
19	93.551	94.098	225	224	137	3	0.75
20	93.8	99.402	116	108	1401	9	2.25
21	94.154	94.67	48	48	129	3	0.75
22	94.661	95.135	48	48	119	14	3.5
23	98.05	98.685	46	46	159	83	20.75
24	98.05	98.565	7	10	129	4	1
25	98.556	99.126	20	23	143	7	1.75
26	99.119	99.66	27	29	135	1	0.25
27	99.788	102.916	100	92	782	77	19.25
28	100.001	105.609	11	15	1402	6	1.5
29	104	104.659	47	47	165	4	1
30	104.661	105.289	47	47	157	6	1.5
31	105.28	105.94	323	335	165	7	1.75
32	105.281	105.885	47	47	151	0	0

^a^ Each point cloud consisted of 4000 laser shots.

^b^ Point cloud 1 was acquired during lifting of the drill. All other point clouds were acquired during descent.

^c^ Numbers in parentheses indicate derived values after exclusion of Spectral Type L385_d_complex features that were only found near 85.5 m and were ascribed to an impurity.

Spectral data from the 1039 points showed a distinctive spectral pattern only near 84.9 m. This was very close to the depth at which new drilling into the borehole took place. We deemed this as a possible impurity and, if the 239 points from this region are removed from the bulk analysis, we obtain a corrected bulk hit rate of 0.6%. As can be seen from examination of Table 1, the actual rate varies from point cloud to point cloud. Two of the point clouds had zero points of interest (out of a 4000 laser shots), whereas one of the point clouds had a hit rate of 2.65% (this point cloud did not have any of the possible impurities in it). No obvious correlation was noted between post-spacing and observed hit rate. Our results suggest that distribution of fluorescent materials in the ice is not uniform and that, on average, many locations need to be interrogated at the 100 μm scale to detect fluorescent materials.

It should be noted that the example in Fig. 3 (from point cloud 17), taken at a depth range of 93.549–94.161 m and a rotation orientation of 48 degrees relative to magnetic N, had 50 identified features. In contrast, a similar transect (point cloud 18) at nearly the same depth range, from 93.549 to 94.217 m, but at a rotation orientation of 33 degrees relative to magnetic N, had only four identified features. With a borehole diameter of 11.176 cm and a circumference of 35.11 cm, a difference of 15 degrees corresponds to a distance along the borehole's curved wall of 1.46 cm. Thus, even at the same depth, a slightly different track, and small cm-scale horizontal distance, can provide different results (Fig. 12). This observation had previously been noted for extracted and laboratory-scanned sections of WAIS divide and GISP2 ice samples by Rohde (2010); here, we note a similar observation *in situ*.

**FIG. 4.**
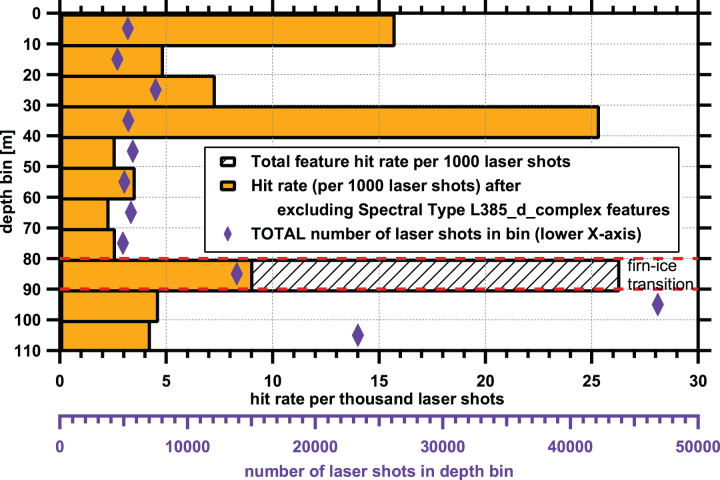
Hit rate frequency per 1000 laser shots as a function of depth. Shaded bars show hit rates of all spectral types, orange colored bars show hit rate if data from the putative impurity, Spectral Type L385_d_complex, is excluded. The purple diamonds and lower *x*-axis show the number of laser shots fired in each depth bin. The zone with the firn-glacial ice transition (80–90 m) is shown by red dashed lines. Color images are available online.

Figure 4 shows the distribution of identified points of interest by 10 m depth bins. It should be noted that more point cloud data (more laser shots) were acquired at deeper locations; the overall number of laser shots in each depth bin is indicated in Fig. 4 as purple diamonds. In general, the number of points of interest per 1000 laser shots is relatively constant from 0 to 105 m, but with higher frequencies in the 80–90 m depth bin, the 30–40 m depth bin, and from the surface to 10 m depth. From visual inspection of the borehole images, the firn-ice transition was in the 80–90 m depth range.

The reason for the significantly elevated hit rates at 30–40 and 80–90 m is unclear. We also note an enhancement of hit rate frequency toward shallower depths. From our down-borehole imaging, we noted a melt lens at 3.28 m depth that we interpret as resulting from the surface melting in summer of 2012 (McGrath *et al.*, 2013). Our most shallow scan (Point cloud map 1, Table 1) was just below that level and that particular scan did not have a higher than normal hit rate. However, it also should be noted that the 1889 melt period, which corresponds to roughly 58 m depth, did not correspond to an increased hit rate in the 50–60 m depth bin (depth correlation adapted from GISP2 data from Mayewski, 1999). The borehole was previously drilled in 2017 to study the physical structure of the ice, and at that time, no specific organic matter decontamination procedures were taken. The 80–90 m-depth bin contains the 85 m maximum depth attained by the Baker team in 2017; thus, if any materials went to the bottom of the borehole, they would be emplaced at this depth. In addition, although we worked in a manner to reduce introduced contaminants, our drilling efforts to 110 m depth before scanning may also have introduced organic matter contamination, particularly at shallower depths that were the most frequently traversed during the drilling operation. However, we note that the sharp zone containing the Spectral Type L385_d_complex at 85 m (see Section 3.3 for Spectral Type naming convention) contaminant suggests that although this material was indeed emplaced at some point, it remained localized even after repeated drill ascents and descents. This suggests that our drilling and scanning operations did not affect material emplacement.

One possible explanation for the variable hit rates by depth bin is that the deposition of material has changed over time. The depth bin containing the firn ice boundary at 80–90 m corresponds to material deposited approximately 205–245 years ago. The age values are extrapolated and adjusted by using data from the work of Mayewski (1999); Yung *et al.* (2007) found that ice at 94 m corresponds to ice deposited 265 years ago as measured in 2007. Anthropogenic inputs increased after the industrial revolution; as an example, the amount of polycyclic aromatic hydrocarbon (PAH) molecules detected in Greenland ice cores increases significantly due to anthropogenic inputs starting from 1930 to present (Kawamura *et al.*, 1994; Gabrielli and Vallelonga, 2015), which we estimate corresponds from ∼44 m depth in our borehole to the surface (adapted from GISP2 data from Mayewski, 1999). In particular, fluorescent airborne microplastics could be responsible for some of the detected signals at shallower depths, and recent studies have shown that microplastics are present in arctic snow samples (Bergmann *et al.*, 2019); plastic production has increased from an annual production of 2 MT in 1950 to 380 MT in 2015 (Geyer *et al.*, 2017), which would correspond to depths in our borehole of ∼38 and 2 m below the surface, respectively. Another factor is potential degradation of some of the chemical signals with increasing depth; analysis by coupled gas chromatography-mass spectrometry of snow pit samples at Summit Station by Jaffrezo *et al.* (1994) suggested that certain PAH molecules degraded with time and depth. Yet another possibility is the difference in measurable optical properties between glacial ice and firn. Differences in firn and glacial ice physical characteristics such as reflective surfaces and air bubbles may promote or hinder detection of ice features near the borehole surface (*e.g*., reflective or scattering firn surfaces may make organic matter detection by this method easier compared with more compressed ice) (Rohde, 2010). For firn ice, the higher amount of scattering from bubbles and other unconsolidated boundaries can create an effective interrogation volume that is brightly illuminated nearer the surface; this also increases the volume of fluorescence emission that can be received (Rohde, 2010). In contrast, glacial ice may have longer pathlengths (light penetration can travel deeper and straighter into the “clearer” ice) before a fluorophore is detected; only fluorophores that lie directly in the beam path will be illuminated (Rohde, 2010; Eshelman *et al.*, 2019).

### 3.2. Multipoint features

Of the 1039 identified points of interest, 308 (30%) were solitary features composed of a single point where consecutive laser shots did not show a significant signal. The majority of points were associated with one or more consecutive shots and had spectrally related signals. We identified 80 clustered features, listed in Supplementary Table S2 by depth from the surface. Many of these features had only a small number of consecutive points: 38 were composed of 2 consecutive points, 18 of 3 consecutive points, and 7 of 4 consecutive points. However, some had a large number of associated points—entry 52 was a feature composed of 237 consecutive points. We interpreted consecutive spectrally similar laser shots as large extended features; the spectral similarity of these provided corroborating evidence that the measured signals are not random spikes in the data. However, due to the varying laser post-spacing in the point clouds, a larger number of consecutive points does not correspond to a larger spanned distance. Supplementary Table S2 lists the maximum estimated size of the features.

We calculated the maximal estimated apparent size by assuming that the laser was scanning in a straight line with uniform spacing and that the size extended almost one post-spacing to the neighboring post just before and just after the feature along the line. A potential overestimate of >10.6 mm could occur in cases where the shot post-spacing is 5.321 mm—this overestimate would occur in the case where the detected laser shot was right at the margin of an extended feature but our estimate extended to the next posting.

**FIG. 5.**
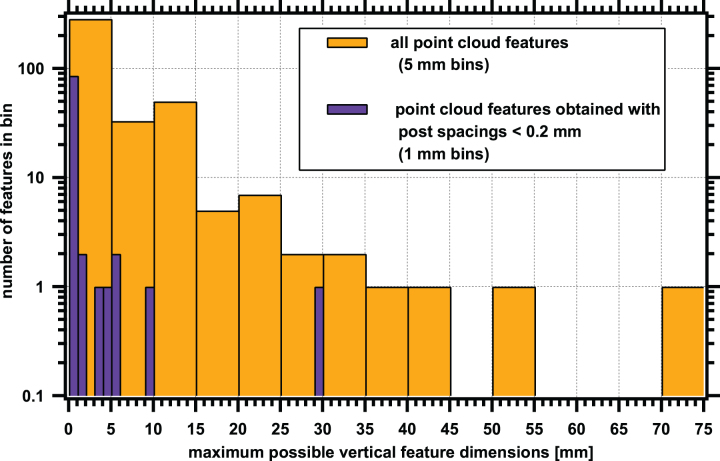
Distribution of estimated size of features identified from point cloud measurements. The size estimate is based on laser spot spacing and the number of points detected (see Section 3.2). The entire dataset of 388 total features (this includes both multipoint features and single points of interest) is shown in orange. For a single feature with the largest post-spacing (5.321 mm for Point Cloud 5), a single point could span 10.642 mm before detection by a second laser shot. Thus, a single point from this Point Cloud could indicate a feature up to 10.6 mm wide and would go into the 10–15 mm bin. A subset dataset (purple bars) represents the data that only have higher resolution post-spacing <0.2 mm. In that case, a single pixel feature would only go into the 0–1 mm bin. Despite the post-spacing variation, both distributions show a skew toward smaller features. Note the log scale on the *y*-axis. Color images are available online.

Given this caveat, the binned distribution of maximally estimated size of all the features in the point cloud data is shown in Fig. 5. For single-point features, the maximum size was estimated to be 2 × the post spacing—it is likely that this is an overestimate based on data from our higher resolution raster map data (see Section 3.4). Due to the variable shot spacing mentioned earlier, only some of the higher-resolution data could be placed in bins smaller than the 10–15 mm bin. Even with this caveat, a clear drop off in larger-sized features can be noted in the graph. There are 20 features (out of 388, thus 5%) larger than 15 mm. The largest feature by distance spanned was 74.5 mm, entry 38 (Supplementary Table S2, detailed spectra of points for this entry shown in Supplementary Fig. S1), which consisted of 13 consecutive spectrally similar shots with a 5.3 mm spacing. In Fig. 5, we also show the data from post-spacings <0.2 mm by using 1 mm bin sizing. Even with this restricted dataset, both distribution patterns in Fig. 5 suggest a skew to smaller-sized features.

The signal intensities at the λ_max_ spanned over four orders of magnitude (Supplementary Data S3 and Supplementary Fig. S2). Weak signals (<2000 instrument counts) predominate over stronger ones. For example, 70% of all points of interest in the point cloud dataset are within 3000 instrument counts of the background, and 95% of all points are below 20,000 background-subtracted instrument counts. No clear correlation between average signal intensity and estimated feature size was noted. However, one of the largest features, entry 77 in Supplementary Table S2, had considerably higher intensity than most other signals. It alone accounted for a large percentage of the overall signals detected above 20,000 counts (see signal intensity distribution plot in Supplementary Fig. S2). The average signal intensities of the point cloud dataset as well as the raster map dataset followed a rough power law, with many features with low average signal intensity and only a few features with high signal intensity (Supplementary Fig. S2).

The rough distribution of signal intensities has important implications for future scanning instrumentation or instrument modifications. For the point cloud dataset, if the instrument sensitivity was decreased so that only peaks above the 5000 count level were considered significant, then only 10% of the signals would be detected; the corresponding hit rate would also drop by an order of magnitude.

### 3.3. Spectral diversity of selected points

**FIG. 6.**
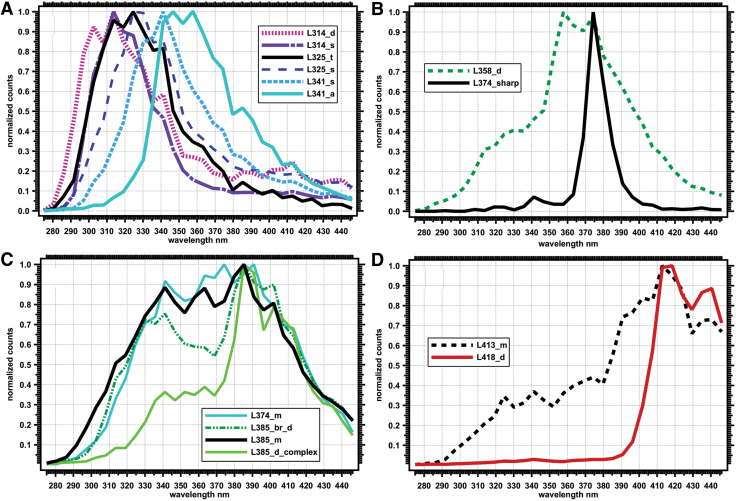
Representative examples of normalized spectra of identified Spectral Types grouped from low to high emission wavelengths. **(A)** Spectral Types with λ_max_ < 350 nm: L314_d, L314_s, L325_t, L325_s, L341_s, L341_a (Table 3). **(B)** Spectral Types with one or two peaks with λ_max_ between 350 and 380 nm: L358_d, L374_sharp. **(C)** Spectral Types with multiple peaks with λ_max_ between 340 and 410 nm: L374_m, L385_br_d, L385_m, L385_d_complex. **(D)** Spectral Types with λ_max_ > 410 nm: L413_m, L418_d. λ_max_, lambda max. Color images are available online.

The selected points from the point cloud data were classified into several Spectral Types based primarily on λ_max_ as well as on fluorescence emission pattern. We show representative spectra in Fig. 6 and characteristics and occurrence frequencies of these Spectral Types in Table 2 in order of increasing λ_max_. We used a naming scheme based on position of the λ_max_ to the nearest nm, followed by a descriptor of the peak shape or pattern position. For example: “s” = single peak, “d” = double peak, “t” = triple peak, “m” = multiple peaks, and “complex” indicating a complex pattern. We designate “sharp” to indicate a peak that is narrower in comparison to the other spectral signatures and “br” to indicate λ_max_ peaks that appear broader than the other spectral signatures. We also use “a”= asymmetrical for peaks that are not symmetrical about the λ_max_. Many of these descriptors are subjective and based on the current set of spectral signatures. Additional descriptions and diagnostic spectral features are presented in Table 2.

**Table 3.** List of Features Identified in the Selected Detailed Map

Region ID	Spectral type	Map location (*x*,* y*)^a^	Lambda max (nm)	Average intensity (counts at lambda max)	Area (pixels)	Area (mm^2^)	Maximum dimension (mm)	Aspect ratio (w:h)^b^	Orientation clockwise from horizontal^b^	Average intensity per pixel per region area
1	L341_s	(4.5, 37.8)	341.2	652	15	0.15	0.81	1.6	−49	43
2	L325_t	(3.9, 37.2)	324.6	4693	44	0.44	0.98	1.4	−43	107
3	L385_m	(2.9, 34.3)	385.3	1448	24	0.24	0.60	1.4	−90	60
4	L385_m	(6.5, 28.7)	385.3	681	30	0.3	1.10	2.0	−47	23
5	L385_m	(5.6, 28.1)	385.3	738	90	0.9	1.79	3.1	−58	8
6	L385_m	(6.4, 27.9)	385.3	1057	81	0.81	1.46	1.4	−45	13
7	L341_s	(10, 26.8)	341.2	2435	20	0.2	0.91	1.8	−42	122
8	L385_m	(5.6, 26.3)	385.3	562	11	0.11	0.71	1.6	−45	51
9	L325_s	(9, 25.9)	324.6	491	38	0.38	1.31	2.1	−46	13
10	L325_t	(8, 26)	324.6	316	36	0.36	1.29	2.9	−85	9
11	L385_m	(7.3, 25.6)	385.3	743	39	0.39	1.21	3.4	−78	19
12	L325_t	(5.1, 24.9)	324.6	720	6	0.06	0.40	2.0	−90	120
13	L374_sharp	(1.4, 10.8)	379.8^c^	1916	9	0.09	0.40	1.8	−56	213
14	L385_m	(5.2, 9.2)	385.3	794	73	0.73	1.53	2.3	−69	11
15	L385_m	(4.4, 8)	385.3	588	108	1.08	1.70	1.2	0	5
16	L385_m	(4.4, 7.2)	385.3	641	46	0.46	1.28	1.4	−47	14
17	L385_m	(5.8, 6.5)	385.3	389	34	0.34	0.84	2.2	70	11
18	L374_m	(1.4, 4.1)	374.3	569	16	0.16	0.74	2.5	−77	36
19	L325_t	(2.6, 2.6)	324.6	2677	15	0.15	0.50	1.4	−77	178

^a^ Map coordinates are given in mm relative to lower left corner (0, 0) in Fig. 9. Each pixel is 0.1 mm.

^b^ These values are after spatial adjustment in the *z*-direction to account for the true orientation and dimensions.

^c^ For this particular feature, the lambda max was at 379.8 nm, and the intensity at 379.8 nm is recorded here.

**Table 2.** List of Spectral Types Identified in the Point Cloud Dataset

Spectral type name	Additional diagnostic spectral features	Total no. of features	Percent of total features (excluding L385_d_complex)	Frequency of occurrence (hit rate per 1000)^a^	Frequency in firn zone (<80 m, hit rate per 1000)^a^	Frequency in firn-glacial transition zone (80–90 m, hit rate per 1000)^a^	Frequency in glacial zone (>90 m, hit rate per 1000)^a^
L314_d	Lambda max 313.7 nm, strong shoulder at 302.6 nm.	19	1.8 (2.4)	0.148	0.182	0.505	0.057
L314_s	Lambda max 313.7 nm, little to no shoulder at 302.6 nm.	1270	12.2 (15.9)	0.992	2.707	0.072	0.100
L325_t	Lambda max at 324.6 nm, shoulders at 308.1 and 341.2 nm with diagnostic dips at 319.1 and 335.7 nm.	150	14.4 (18.8)	1.172	1.319	0.794	1.154
L325_s	Lambda max at 324.6 nm, little to no shoulder at 313.7 nm.	20	1.9 (2.5)	0.156	0.182	0.289	0.114
L341_s	Lambda max at 341.2 nm, symmetric peak.	44	4.2 (5.5)	0.344	0.068	0.289	0.527
L341_a	Lambda max at 341.2 with sharp rising asymmetric peak; 324.6 nm <20% signal.	9	0.9 (1.1)	0.070	0.205	0	0
L358_d	Double peak at 357.7 and 374.3 nm.	56	5.4 (7.0)	0.438	0.091	3.753	0
L374_sharp	Single sharp peak at 374.3 or 379.8 nm; usually <10 pixels in spatial extent.	5	0.5 (0.6)	0.039	0.091	0	0.014
L374_m	Broad rise to complex pattern with peaks at 341.2, 363.3, 374.3, and 390.8. Peaks at 374.3 and 390.9 nm nearly equal.	27	2.6 (3.4)	0.211	0.500	0.217	0.028
L385_br_d	Broad doublet with lambda maxes at 341.2–385.3 nm; no prominent peaks in between.	28	2.7 (3.5)	0.219	0.091	0	0.342
L385_m	Broad complex with peaks at 341.2, 363.3, 385.3, and 390.8 nm and a shoulder at 401.9 nm. Peak at 363.3 nm generally higher than shoulder at 401.9 nm; peak at 374.3 nm absent.	196	18.9 (24.5)	1.531	1.365	2.959	1.354
L385_d_complex	Broad rise to peaks at 341.2, 363.3, 385.3 nm, and 390.8 nm and shoulder at 401.9 nm. Peaks at 385.3–401.9 nm are 2 × higher than peaks at 341.2–363.3 nm.	239	23.0 (0.0)	1.867	0	17.249	0
L413_m	Broad rise to multiple peak features with lambda max at 412.9 nm.	65	6.3 (8.1)	0.508	1.092	0.144	0.214
L418_d	Sharp rise to peaks at 412.9–418.4 and 435–440.5 nm. Clear dip from 423.9 to 429.4 nm.	54	5.2 (6.8)	0.422	0.205	0.072	0.627

^a^ Frequency refers to the total number of points of interest with that Spectral Type in the entire dataset versus the total number of laser shots.

We can use the spectral properties of the λ_max_ to infer some properties of the molecular structure and complexity, or at least the electronic structure of the fluorescing material (Bhartia *et al.*, 2008). In general, at low excitation wavelengths, single-ring aromatic molecules with limited conjugating or electronic-modifying functional groups have fluorescence emission maxima <300 nm. Larger conjugated or fused aromatic systems with three or more rings have fluorescence emission maxima >400 nm. Bicyclic systems, including the indole ring of the amino acid tryptophan, have fluorescence emission maxima between 325 and 375 nm.

**FIG. 7.**
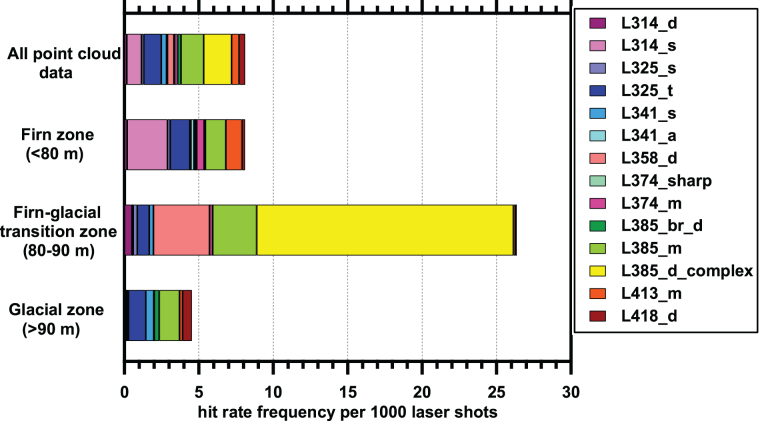
Frequency of the different spectral types at varying depths. From the surface to the firn-glacial transition zone, points of Spectral Type L314_s are common, whereas deeper than 90 m, they are rare. In contrast, points of Spectral Type 341_s are more common in glacial ice. Color images are available online.

**FIG. 8.**
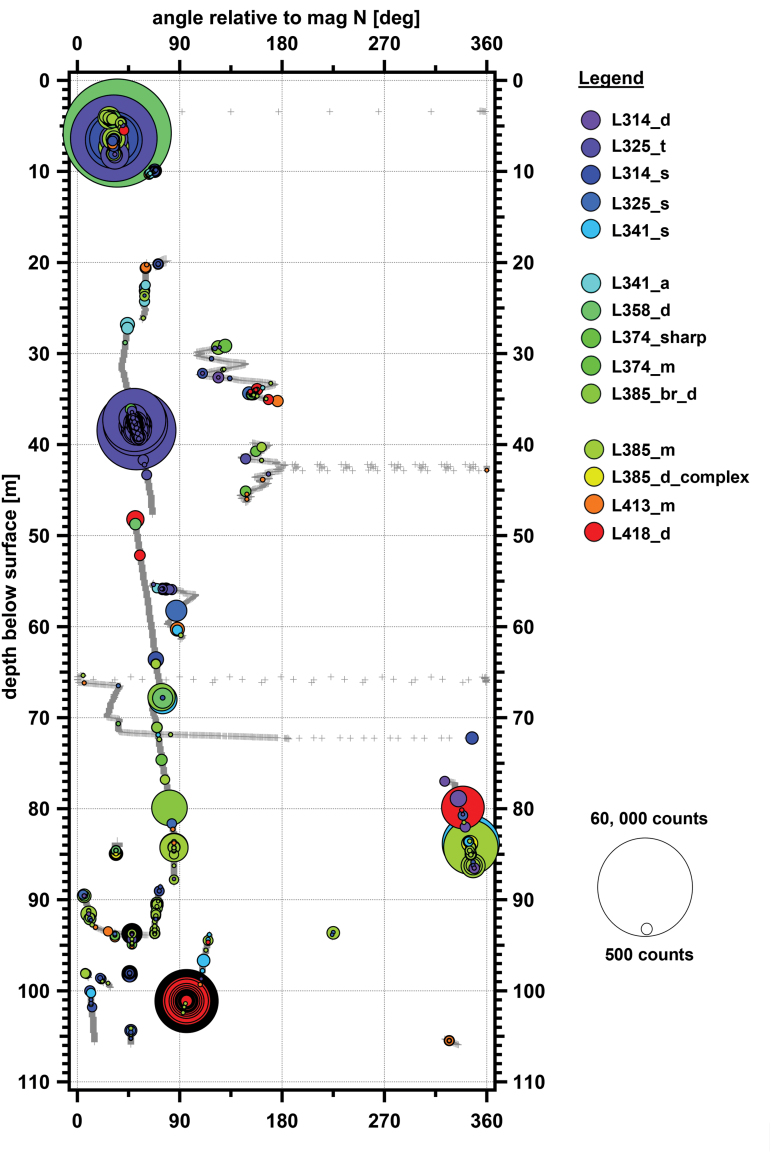
Plot of point cloud data in borehole coordinates. Cylindrical coordinates with rotation relative to magnetic north along *x*-axis, and depth in borehole along *y*-axis. Gray crosses indicate locations where laser data were acquired but signals above the noise level were not detected. Colored bubbles indicate detected features, with color based on λ_max_ of detected feature from purple (314 nm) to red (418 nm). Size of the bubble is based on background-subtracted signal intensity at the λ_max_. Color images are available online.

Figure 7 shows a graphical distribution of the Spectral Types across different depths. Points of some Spectral Types have a higher frequency in one depth bin compared with other bins, which suggests some type of change in deposition history, fractionating mechanism, or *in situ* modification. For example, Spectral Type L314_s (pink colored zones in Fig. 7) was mostly found in the upper firn ice zone, whereas Spectral Type L341_s was mostly found in the glacial ice zone (older ice), although both Spectral Types were found throughout the borehole column. Of note is the Spectral Type L385_d_complex spectral type. This Spectral Type was only found in the 80–90 m bin, was specifically localized to 83.82–84.94 m, and was found in two Point Clouds at that depth: Point Cloud 12 and 14 (these can be seen as the yellow-colored bubbles in Fig. 8).

The spectral response of L385_d_complex appears similar to Spectral Type L385_m; unlike the localized L385_d_complex, Spectral Type L385_m is found throughout the borehole. The ratios of intensities for peaks in the spectrum of L385_d_complex are constant, with the signal response at 385.3 nm roughly 3 × the response at 341.2 nm. In contrast, peak intensity ratios of corresponding peaks in the spectrum of Spectral Type L385_m vary. The constant ratio between the peaks of L385_d_complex suggests a uniform spectral composition in all the identified points of interest, whereas the varying peak ratios in L385_m spectra could indicate varying compositions among the different points of interest of that Spectral Type. From this characterization, we suggest that although L385_d_complex could be spectrally related to L385_m, it is a distinct and localized subpopulation of features—consistent with (but not diagnostic for) the possibility of this material being an introduced impurity. The localization at the depth where previous drilling efforts halted is also consistent with an introduced impurity.

During our analysis of Spectral Type occurrence frequency, we present frequency numbers in Table 2 that both include and exclude the putative impurity Spectral Type L385_d_complex. After excluding Spectral Type L385_d_complex, most of the selected points from the point cloud data are Spectral Type L385_m (green zone in Fig. 7), which makes up almost 25% of all the selected points and is distributed in both shallow firn and deeper glacial ice. The second most common is Spectral Type L325_t (blue zone in Fig. 7), and it makes up 18.8% of all the selected points, which are found with similar frequencies in both shallow firn and deeper glacial ice. Spectral Type L314_s (light purple zone), Spectral Type L413_m (orange zone), Spectral Type 358_d, Spectral Type 418_d, and Spectral Type 341_s comprised 15.4%, 8.1%, 7.0%, 6.8%, and 5.5% of all identified points of interest, respectively. From the plot, it appears that Spectral Type L418_d is found predominantly in the lower glacial parts of the borehole; however, below 90 m, Spectral Type L418_d is only found in two features in the point cloud dataset, one of which is a large and bright 43-point feature (entry 77 in Table 2) and the other is a relatively dim single-point feature. Spectral Type 358_d is found primarily in the transition zone at 80–90 m, although there are other rare occurrences in firn, but not glacial ice. The other Spectral Types listed in Table 3 and shown in Fig. 7 (L314_d, L341_a, L374_sharp, L374_m, L385_br_d) are all minor components and combined make up <15% of all identified points.

### 3.4. Borehole map from point cloud dataset

Figure 8 shows a consolidated view of all the point cloud data to create a partial map of the borehole by point intensity. This is in a form of cylindrical projection, with rotational degrees from magnetic N along the *y* axis and depth along the *x*-axis. The axes in Fig. 8 are not to the same scale; with a diameter of 11 cm (4.4 inches), the actual borehole circumference is 0.35 m and would not be easily visible at this graphic scale in an undistorted image. Gray crosses show locations where the laser fired, but no significant return fluorescence signal was detected. Colored bubbles show locations of identified points of interest; the color of the bubble in the graphic is based on the Spectral Type of the signal, and the size of the bubble is related to the background-subtracted signal strength at the λ_max_. Deviations from straight tracks parallel to the *y* axis show where the instrumented-drill combination rotated during its path down or up the borehole.

From initial inspection, the absolute intensity of the peak is not affected by the depth. However, as previously noted, there are a few intense points of interest of Spectral Type L418_d found above 80 m, whereas below 90 m they are some of the most intense points of interest encountered, represented by large nested circles in Fig. 8 at ∼102 m depth. If these Spectral Type L418_d points are removed (and they may be one single feature since many of the signals are consecutive returns from the same point cloud transect), then there is a slight trend of a decrease in signal intensity for signals in glacial ice.

### 3.5. Map analysis

**FIG. 9.**
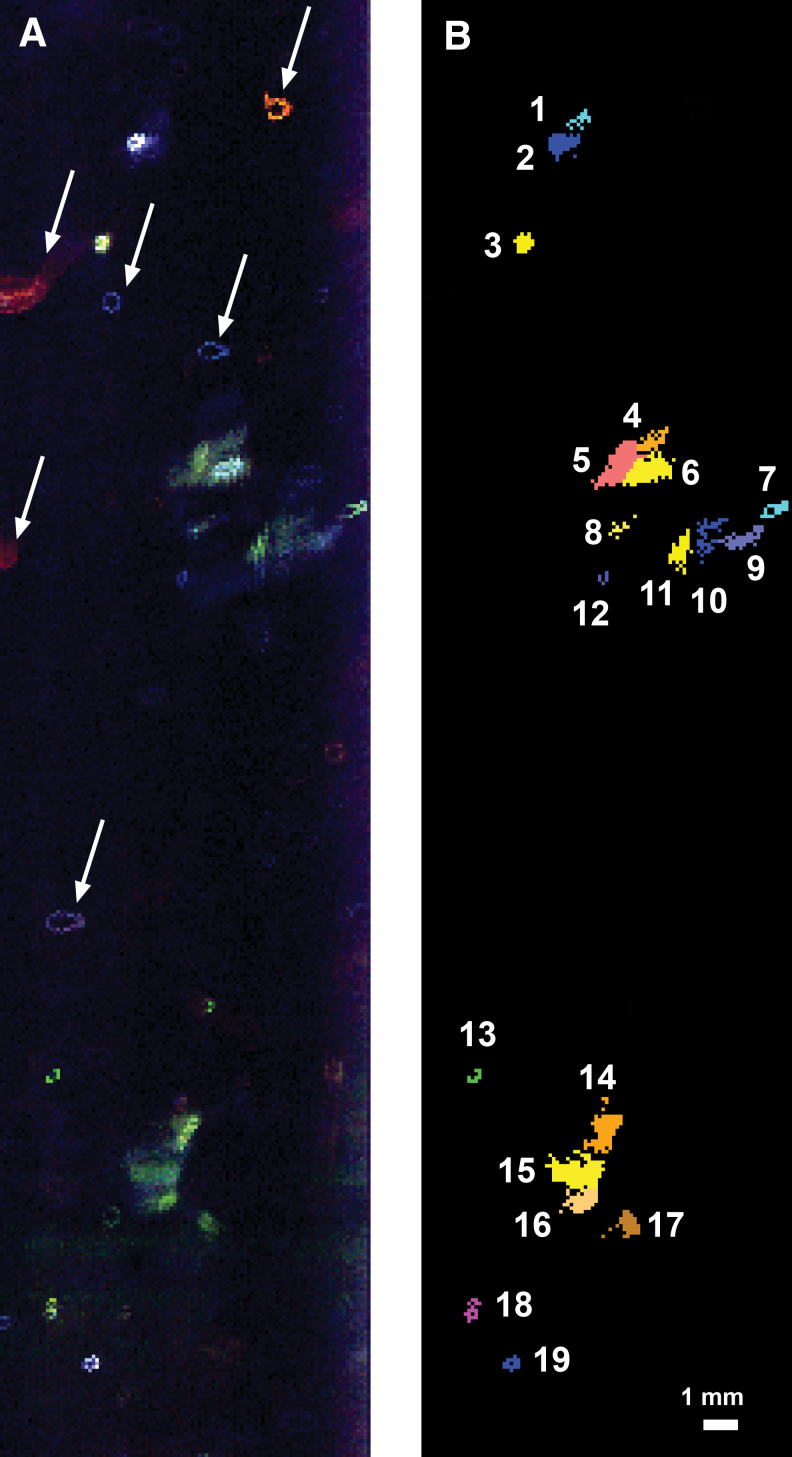
Different views of *in situ* fluorescence data acquired from the ice borehole from Map 1 at 93.800 m depth below the surface. **(A)** Contrast-adjusted stretch of RGB (412.9, 385.3, and 313.7 nm). White dotted arrows indicate artifacts, mostly ringlets, likely on the optical window that appear in multiple maps. **(B)** Annotated ROIs with colored squares indicating the 19 brightest features in this scene. Color key: Blue corresponds to Spectral Type L325_t features, Cyan is Spectral Type L341_s, Yellow/Orange/Melon is Spectral Type L385_m, Green is Spectral Type L374_sharp, and Magenta is Spectral L374_m. Feature labels relate to Table 4 and text. Scale of all scenes as shown in panel B. The top margin of the map image is toward the top of the borehole. The left and right edges are 41.3 and 51.7 mm along the circumference of the borehole from magnetic N, respectively. The bottom edge of the map is located at 93.7987 m depth from the surface, whereas the top edge is located at 93.7575 m depth. ROI, region of interest. Color images are available online.

More than 26 maps were collected spanning depths of 10–106.7 m. For detailed analysis, we selected Map 1, a 1 × 4 cm map acquired at a lower depth of 93.8 m and a rotation of 53 degrees shown in Fig. 9 (these coordinates are for the lower right corner of the collected map). The exact dimensions were 1.05 × 4.13 cm. Map 1 had a shot spacing of 0.1 mm (100 μm) and dimensions of 105 columns × 413 rows. This map had a variety of clearly distinguishable bright features. Supplementary Data S4 has bias-corrected spectral data for all of Map 1 listed by pixel coordinate.

The collected multiband data were visualized and analyzed by ENVI (Harris Geospatial Systems). Figure 9A shows an ENVI plot of the data using 412.9, 385.3, and 313.7 nm data, where the stretch of the visualization was set so that signals 3 × the standard deviation above an average background across the map are rendered visible. In the figure, multiple artifacts appear as ringlets; these are likely impurities on the window and are observed across multiple maps in the same relative position. Figure 9B shows a set of selected ROIs corresponding to pixels above the limit of detection (LOD = 3 × noise) in at least one of the instrument bands. The ROIs in Fig. 9B are colorized based on their assigned Spectral Type.

**FIG. 10.**
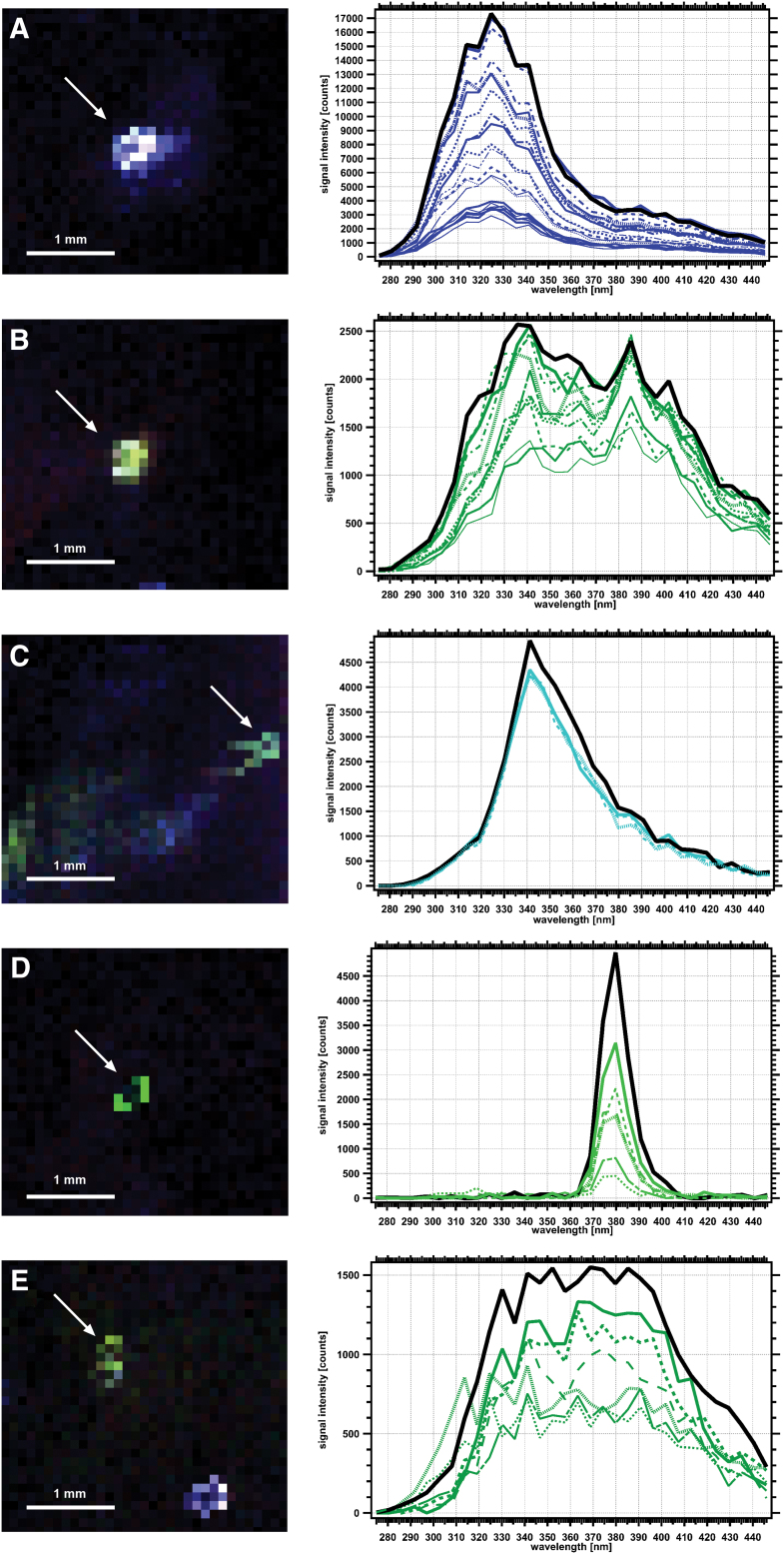
Detailed view of five selected ROIs from Map 1 taken at 93.8 m depth shown in Fig. 9 and corresponding extracted spectra in corrected instrument counts for region-of-interest pixels with peaks 10 × above interpolated noise. Each line shows extracted spectra from a given pixel plotted on an intensity scale extending to the maximum fluorescent maxima values for that ROI. **(A)** ROI 2, of Spectral Type L325_t, centered at (3.9, 37.2 mm). **(B)** ROI 3, of Spectral Type L385_m, centered at (2.9, 34.3 mm). **(C)** ROI 7, of Spectral Type L341_s, centered at (10, 26.8 mm). **(D)** ROI 13, of Spectral Type L374_sharp, centered at (1.4, 10.8 mm). **(E)** Feature 18, of Spectral Type L374_m centered at (1.4, 4.1 mm). In this same view, there is a circular region at lower left that is of Spectral Type L325_t (spectral data not shown). Coordinates given in *x*-offset and *y*-offset from origin of Fig. 9 map at lower left. Scale in all plots as shown. White arrows indicate selected regions. See Fig. 9 for map location of these features and Table 4 for further details. Color images are available online.

Figure 10A–E shows a detailed view of selected spectrally diverse ROIs from Map 1, including several of the brightest regions. These are shown next to a stacked plot of spectra from the indicated region that are above the limit of quantification ( = 10 × noise) in at least two wavelengths. From the plotted spectra, there is minimal spectral variation of the individual pixels composing the region—the regions are spectrally uniform.

### 3.6. Analysis of identified ROIs in map from 93.8 m depth

A list of the identified ROIs from Map 1 is presented in order of increasing depth below the surface in Table 3. The spectral characteristics were defined based on similarities with the spectral classifications described in Table 2. No new Spectral Types were observed during the analysis of Map 1—all the detected features could be placed in the Spectral Type classification scheme presented in Table 2. Map 1 ROIs had a large relatively large spectral diversity; of the 14 Spectral Types noted in Table 2, 5 of these were observed in Map 1. Supplementary Data S5 lists all the pixels extracted from all the ROIs in Map 1.

Several of the ROIs with similar emission spectra were characterized as separate regions with perceived morphological breaks with other ROIs. This includes the cluster of Features 4–6 and Features 14–16 (Fig. 9). Since they are proximal to each other and spectrally related, they could be perceived as part of the same feature, if the connections were not visible due to their depth in the ice. In the work of Eshelman *et al.* (2019), we reported that the pathlength of the WATSON instrument is up to 2 cm deep in clear bubble-free laboratory ice. The exact pathlength inside the boreholes is not known, but we assumed roughly <2 cm due to bubbles; thus, features and connections of extended features that are deeper than this pathlength would not be observed in our map and could create an apparent visual disconnection.

The areal size of the ROIs ranged from 0.06 to 1.08 mm^2^, with an average area of 0.4 mm^2^. If ROIs 4–6 were combined, they would make an extended region with a spatial area larger than 2 mm^2^ and if ROIs 14–16 were combined, they would make an extended region with a spatial area larger than 2.3 mm^2^ (the extended regions would be larger in area than the summed individual regions since some apparently empty pixels lie between the regions).

The maximum linear dimensions of the ROIs are also indicated; in general, the largest features in Table 3 are less than 2 mm across. If ROIs 4–6 were combined, they would make an extended region 2.61 mm across; whereas if 14–16 were combined, they would make an extended region 3.34 mm across. If the extended regions and remaining isolated ROIs were binned into 1 mm size bins according to the maximum dimension, the resulting binning and number of entries would be: 0–1 mm: 10; 1–2 mm: 3, 2–3 mm: 1, and 3–4 mm: 1. This is very similar to the distribution presented in Fig. 5 for the high-resolution (post-spacing <0.2 mm) data. Thus, the distribution of the maximum dimension of the ROIs in Map 1 appears representative of the estimated size distribution of the entire point cloud dataset collected throughout the borehole. The features identified in Map 1 are all at the sub-cm scale. Rare larger features may be possible according to the Point Cloud data in Table 2 and Fig. 5, however we did not observe these large features in Map 1.

After spatial correction, we measured the maximum dimensions as well as the maximum dimension orthogonal to this direction to determine the aspect ratio of the ROIs; most of the features had an aspect ratio between 3.4:1 (oblong) and 1.2:1 (nearly spherical) with an average aspect ratio of 2:1. From the dimension measurements, we also measured the apparent angle or tilt relative to horizontal. In general, for most of the ROIs where an angle could be recorded, regions were tilted roughly 40–70 degrees counter-clockwise to horizontal, as if they were originally spherical objects that were compressed from the upper left. The sole exception is ROI 17, which has a tilt to the upper right.

The intensities of the signals across different ROI were compared by using the background-subtracted signal intensity at relative λ_max_ wavelengths for each region's assigned Spectral Type. The assumption is that regions may have similar fluorescence intensities and similar quantum yield. We determined the average intensity by summing all the background-subtracted intensities at the λ_max_, then dividing by the number of pixels for the overall feature. In Map 1, we found that the pixels with the highest intensity were classified as Spectral Type L325_t, specifically, Features 2 and 19.

### 3.7. Estimate of hotspot frequency in Map 1 data

The total number of pixels in view for Map 1 is 43,365 pixels, which allows us to compare the hit rate frequency with the point cloud data presented in Table 2.

**FIG. 11.**
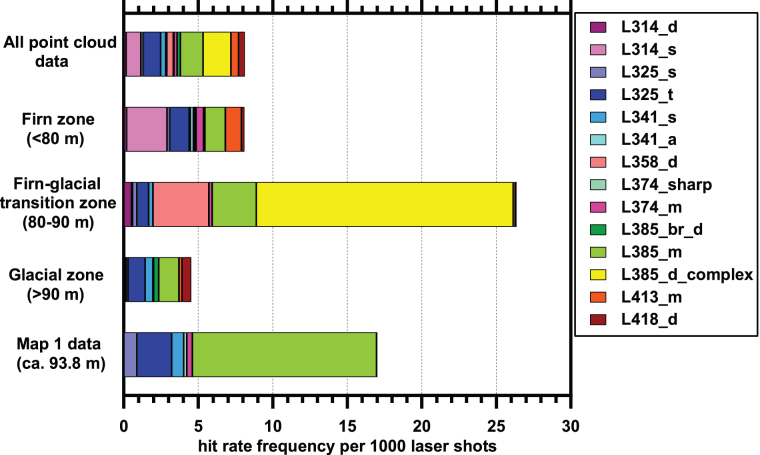
Comparison of Map 1 Spectral Type frequency with frequencies of point cloud data from varying depths (Fig. 4). Map 1 has an elevated number of features, although the absolute signal intensities are lower than for the bulk point cloud data (Supplementary Fig. S2). Color images are available online.

A summary of data in Map 1 as broken down by Spectral Type is in Table 4, with a graphical comparison Map 1 Spectral Types to point to cloud Spectral Types presented in Fig. 11.

Examination of Table 4 shows that by number of regions, most regions in Map 1 are Spectral Type 385_m. Spectral Type 385_m also has the highest spatial areal coverage, with more than 72% of the pixels in the ROIs with this spectral type, but only slightly more than 50% of the total hotspot integrated signals at λ_max_. Thus, in Map 1, the Spectral Type 385_m features are numerous, large, and have low signal intensity. The averaged hit rate (pixels of this Spectral type vs. pixels examined) for this spectral type is 12.4 per 1000, which is roughly 10 × higher than the overall hit rate determined in Table 2 for this Spectral Type below 90 m depth.

In Map 1, only four ROIs of Spectral Type L325_t are observed. They make up <15% of the total hotspot area yet have more than 30% of the total signal. In general, these signals are bright. In Map 1, they have a hit rate frequency of more than 2 points per 1000 laser shots, but in the point cloud data in Table 3, they have an average hit rate of 1.2 points per 1000 laser shots for locations below 90 m; thus, the frequency of these points is only 2 × “typical” expected values. We should recall that Map 1 was selected for its richness of ROIs, thus the hit rate is expected to be above those for bulk point cloud data.

The hit rate for Spectral Type L325_s is enhanced relative to that observed in the point cloud dataset for depths below 90 m, whereas for L341_s it is roughly similar (Fig. 11). For L374_sharp and L374_m, the hit rate is higher than the point cloud data below 90 m; however, these two Spectral Types are rare and only one ROI for each is observed in the map. Thus, Map 1 appears to have a slightly enhanced hit rate for most spectral types, when compared with point cloud data, with an exceptionally high number (10 × ) of signals of Spectral Type L385_m.

## 4. Discussion

### 4.1. Size and distribution and size of hotspots and features

Numerous (178) features were identified in the point cloud data. Most of these were single-point features, although the post-spacing in some point clouds was large enough that a feature as large as 10.6 mm could be detected as a single point. Some larger features were noted, although features larger than 15 mm were rare, comprising <4% of the total number of features. This is in general agreement with our Map 1 data, where all of the ROIs, even after combination of neighboring spectrally similar ROIs, would have dimensions <4 mm. In preliminary analysis of other map data, we saw no evidence of ROIs larger than 15 mm. From the map data presented in Fig. 9, the hotspots appear to have an area of ∼0.4 mm^2^ for the brighter hotspots and they appear as compact <1 mm diameter circular (Features 2, 3) to ring-like (Features 7, 13, 19) features with near-uniform spectral signatures of varying intensity. The aspect ratio (1.2–3.4:1) and angle of apparent compression in Map 1 is consistent with a uniform compression amount and direction of compression across the scene that causes any initially spherical objects to appear slightly out of round, but not completely flat.

**FIG. 12.**
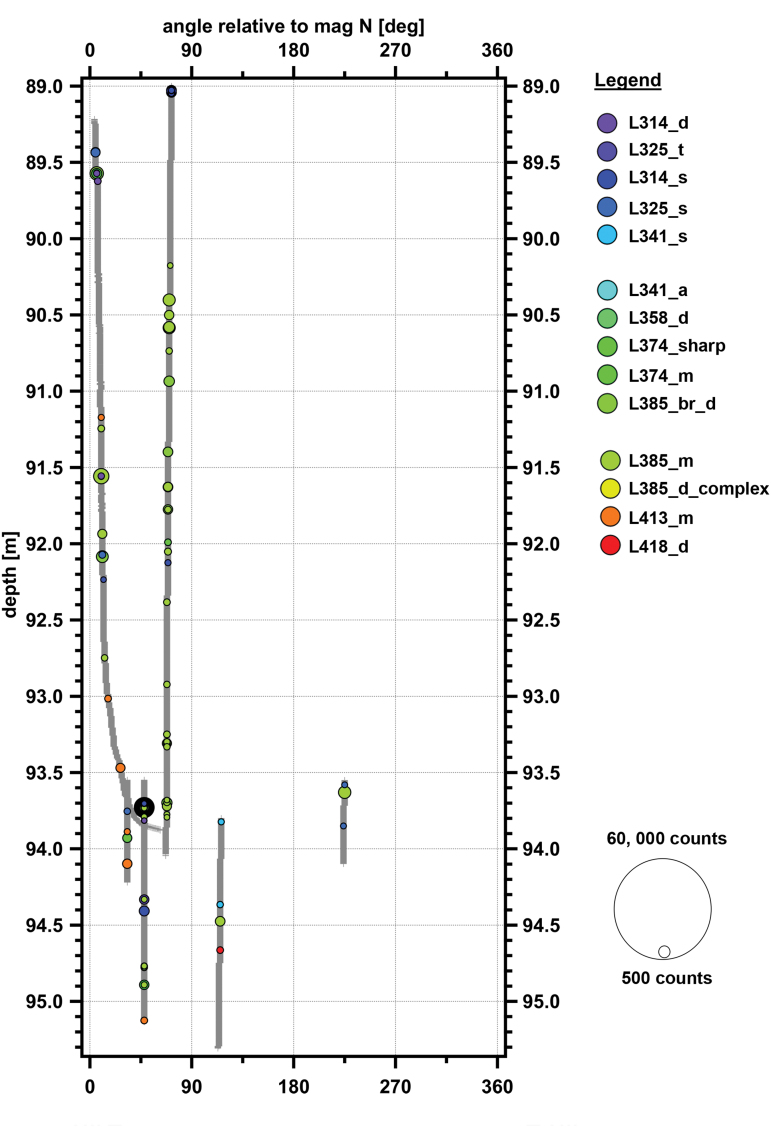
Detailed plot of point cloud data in borehole coordinates near 94 m depth. Features do not line up with other features on parallel tracks, arguing against a consistent layer at a given depth. Legend is same as in Fig. 8. Cylindrical coordinates with rotation relative to magnetic north along *x*-axis, and depth in borehole along *y*-axis. Gray lines indicate locations where laser data were acquired but signals above the noise level were not detected. Colored bubbles indicate detected features, with color based on λ_max_ of detected feature from purple (314 nm) to red (418 nm). Size of the bubble is based on background-subtracted number of instrument counts at the λ_max_. Color images are available online.

There was no clear evidence for layers in the Map 1 data. The point cloud data only provides one dimension; thus, series of consecutive points would look similar to a thick horizontal layer if only the vertical axis were scanned. Some sections of the borehole were scanned with more than one vertical transect, but at a different angle. In many cases, a track with a hotspot at one rotational angle did not show a corresponding hotspot at the same depth but a different rotational angle. This observation is consistent with discrete spots, rather than layers. As an example, Fig. 12 shows a detailed map of *in situ* WATSON scanning of a section of the borehole 92.8 to 95.1 m with multiple point cloud tracks. Detected hotspots in one track are not repeated on another track. This effect had previously been noted by Rohde *et al.* (2008) and Rohde (2010) during laboratory fluorescence scanning of WAIS Divide and GISP2 core sections. It is assumed that the deposition layers at our borehole would be relatively flat lying, so that any distinct spectral layers should be noted by a series of spectrally similar hotspots at nearly the same depth on different tracks. The image in Fig. 12 has been distorted to better show the rotational dimension (*x*-axis in figure) of the borehole. Given the 11 cm diameter of our borehole, a dip of 45 degrees would generate a 0.1 m deviation from one side of the borehole to the opposite side and should still be evident in the graphic.

In general, features do not line up with other features on parallel tracks, arguing against consistent layers or bands at a given depth. Although we saw no evidence of layers from the Map 1 or point cloud data, it is possible that some of the detected broad features in the point cloud data occur in layers, zones, or even spotty confined zones, but to fit our observations, most of these would have to be limited to areas that were scanned with only a single track. The data presented in Figs. 8, 9, and 12 suggest that fluorescent materials such as microbes and organics are not emplaced in evident layers at the fine scales we examined.

With this method and at this fine resolution, the hotspots we observed appear to be stochastically distributed. The sizes varied from 74 mm to a single pixel in the point cloud data, and from 0.4 to 3 mm in the map data. In both datasets, smaller sizes were more prevalent.

In an analysis of 224 nm excitation fluorescent signals along a 350 μm spaced linear track of an isolated and prepared GISP2 ice core (the equivalent of a WATSON point cloud, but with a different excitation wavelength), Rohde *et al.* (2008) showed that both microbes and nonmicrobial aerosols are deposited onto the ice in bursts that are discontinuous on a vertical scale of millimeters to centimeters. This is consistent with our fine-scale results showing that materials are discontinuously distributed both by depth and in borehole rotation.

### 4.2. Constraints on distribution and emplacement mechanisms

Our data allow us to evaluate possible emplacement mechanisms for fluorescent materials that explain our observations. From analysis of our combined point cloud data, the estimated feature size does not appear to vary with depth. Features at the top of the borehole column do not appear significantly larger than those at the base, after the firn-glacial transition, suggesting preservation mechanisms in the ice. This observation from the point cloud dataset is also consistent with preliminary examination of our detailed raster map data—average hotspot feature size does not appear to vary according to depth. These observations suggest that the initial emplacement places a spectrally uniform material at a specific location and size in the near-surface ice, and that the transition from firn to glacial ice does not cause significant migration or transformation.

The observed size of the detected particles constrains the origin and emplacement processes. The maximally estimated size for our largest detected ROI is 74.5 mm (Supplementary Table S2, entry 38, and Supplementary Fig. S1). However, if the points at the ends of the consecutive series of signals are estimated to have hit the outer margin of the extended feature, then the minimum possible size can also be estimated based on post-spacing; the minimum size of the feature in Supplementary Table S2, entry 38 is 63.9 mm. Thus, the estimated size of this feature ranges from 63.9 to 74.5 mm. In general, particulate materials larger than 10 μm (0.01 mm) will settle in a matter of hours from an air suspension. “Heavy dust” is defined as materials <1 mm; some of our detected features are larger than that by nearly two orders of magnitude. Thus, a direct meteoric origin that deposits pure fluorescent organics directly on the surface at these sizes seems unlikely. However, an indirect process, such as an enriched single snowflake with fluorescent material of this size, is possible.

A more likely scenario is that organic material is originally deposited, perhaps as diffuse regions, but is then concentrated into discrete and stochastically located hotspots in the ice at a fine scale. This would happen at the upper surfaces of the ice, as the size of the regions and spectral nature is preserved intact in the ice column. The uniform size may be related to diffusion through the ice, with diffusion occurring quickly at the surface to form an initial ca. 1 mm spot, which is then “locked in” and carried through the firn to glacial ice transition and densification. This may occur at a fine scale that has not been previously observed by bulk analysis. Exact mechanisms are constrained by spectral uniformity, scale that favors smaller hotspots (1 mm being common), favorable energetic requirements, and stochastic emplacement.

### 4.3. Compositional constraints: comparison with laboratory spectra

**FIG. 13.**
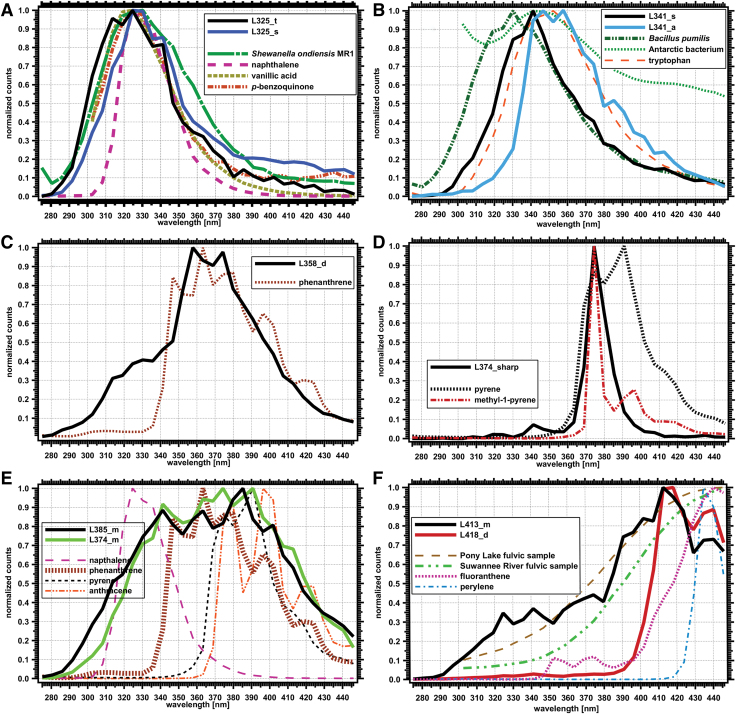
Comparison of identified features with known biological, chemical, and environmental materials. In the figures, spectra isolated from WATSON borehole scanning are in bold lines, whereas laboratory standards are in dotted or dashed lines. **(A)** Spectral Type L325_t (bold black line) and L325_s (bold blue line) with *Shewanella oneidensis* MRI whole cells, naphthalene, vanillic acid, and *p*-benzoquinone. **(B)** Spectral Type L341_s (bold black line) and L341_a (bold light blue line) with *Bacillus pumillus*, Antarctic bacterium isolate (Dieser *et al.*, 2019), and tryptophan. **(C)** Spectral Type L358_d (bold black line) with phenanthrene. **(D)** Spectral Type L374_sharp (bold black line) with methyl-1-pyrene and pyrene. **(E)** Spectral Type L385_m (bold black line) and Spectral Type L374_m (bold green line) with naphthalene, phenanthrene, pyrene, and anthracene. **(F)** Spectral Type L413_m (bold black line) and Spectral Type L418_d (bold red line) with Pony Lake and Suwannee River fulvic acid samples (D'Andrilli *et al.*, 2013), fluoranthene, and perylene standards. Color images are available online.

Spectral Types detected in the Summit borehole are compared with selected laboratory standards of microbial, chemical, and field sample materials in Fig. 13. This figure also compares our data with excitation–emission data in the literature when a 250 nm excitation wavelength data could be isolated (Coble *et al.*, 1990, 2014; D'Andrilli *et al.*, 2013, 2017, 2020) and includes comparisons with natural organic matter mixtures that have a more characteristically complex fluorescent nature. It is important to note that the standard spectra were acquired at room temperature in aqueous or methanolic solution in the laboratory, whereas WATSON spectra were acquired in an ice matrix at roughly −20°C in field conditions; consequently, our measured signals may not overlap exact regions of laboratory standards. Although a rigorous identification is beyond the scope of this article, our identified spectra compare favorably with likely material targets in the polar environment and with previous studies of fluorescent organic matter of various sources (Coble *et al.*, 2014; D'Andrilli *et al.*, 2017).

#### 4.3.1. Shorter wavelength signals

**FIG. 14.**
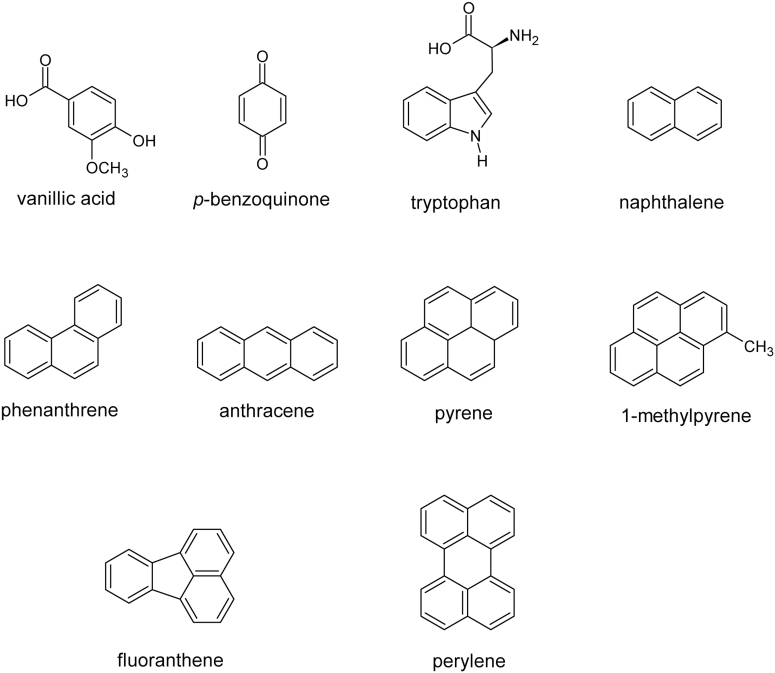
Molecular structures of various laboratory chemicals used as comparison spectra organized in order (left to right, top to bottom) of increasing electronic delocalization and longer λ_max_ wavelength.

Spectral Types L325_t and L325_s have λ_max_ values around 324.6 nm and are similar to whole-cell spectra of bacterial psychrotolerant strains such as *Shewanella oneidensis* MR1 and *Bacillus pumilis* (shown in Fig. 13B). Materials with peaks in this range also include broken-down products of lignins such as lignin phenols (Hernes *et al.*, 2009; Coble *et al.*, 2014); these signatures are similar to those found in glacial Antarctic ice WAIS Divide ice cores (D'Andrilli *et al.*, 2017). The molecular structures of vanillic acid (an example of a lignin phenol) as well as other organic molecules used for spectral comparison are shown in Fig. 14. Naphthalene, a two-ring PAH, also has a similar spectrum with an emission λ_max_ in the same region. The features labeled L341_s and L341_a both have fluorescent spectra similar to whole-cell bacterial cultures and field isolates as well as similarity to the amino acid, tryptophan (Fig. 13B) (Teale and Weber, 1957; Coble, 1996; Eshelman *et al.*, 2019). We, thus, take the suite of Spectral Types with λ_max_ values from 325 to 341 nm as being consistent with microbes or biologically derived materials (including biofilms, see Smith *et al.*, 2016), lignin-breakdown products, and bicyclic aromatic species. This suite of Spectral Types is not consistent with simple benzenes that have shorter fluorescence λ_max_ values <290 nm or higher-order multi-ring aromatic systems that have longer λ_max_ values (*e.g*., phenanthrene) (Fig. 13C). Shorter-wavelength fluorescing Spectral Types, such as L314_d and L314_s (data shown in Fig. 6A), are similar to fluorescence features seen in EEMs data from WAIS Divide ice cores that were ascribed to simple lignin phenols (D'Andrilli *et al.*, 2017; see low excitation and emission wavelength fluorescence example C1 presented in Fig. 2A in that text.).

#### 4.3.2. Longer wavelength signals

The Spectral Types at longer wavelengths >>341 nm are consistent with PAHs or larger aromatic species (see Fig. 14 for molecular structures) and are consistent with spectral signatures that were observed by Rohde *et al.* (2008). These chemical species have been categorized as more complex compared with chemical species that fluoresce at shorter wavelengths from ancient and modern ice cores (D'Andrilli *et al.*, 2017) and can originate from microbial and continental nonmicrobial sources (*e.g*., Pony Lake and Suwannee River fulvic acids).

In general, the emission maxima of Spectral Type L358_d and L374_sharp are above expected values from microbial cells where the spectra may be driven by fluorescence from the amino acid tryptophan and other aromatic amino acids (Coble *et al.*, 2014). Instead, the spectral responses of these longer-wavelength Spectral Types may be derived from more complex materials that may or may not be directly related to microbial processes. The spectral features of Spectral Type L358_d (Fig. 13C) and the sharp Spectral Type L374_sharp (Fig. 13D) are both similar to spectra from PAHs such as phenanthrene, pyrene, and 1-methylpyrene. Figure 13E shows the complex spectrum of Spectral Type L385_m and L374_m as solid lines, and comparison spectra from PAHs already listed including three-ring linear-fused anthracene.

Although none of the spectra are exact matches, alkyl or heteroatom-substituent substitution as well as changes of matrix conditions (water vs. ice, temperature effects) could cause changes to the reference spectra (Johnson *et al.*, 2011). Thus, we can state that the spectral signals are broadly consistent with fluorescence spectra from PAHs, and that they are not similar to the fluorescent signals of microbial cells.

Sources of PAHs for the Greenland ice sheet include the burning and emissions coming from Northern Hemisphere sources (Jaffrezo *et al.*, 1994; Slater *et al.*, 2002; Von Schneidemesser *et al.*, 2008), including boreal forest fires and fossil fuel combustion. Figure 13F shows spectra from Spectral Types L413_m and L418_d. These are compared with Pony Lake fulvic acid, Suwannee River fulvic acid, as well as fluoranthene, a PAH with a chemical structure similar to naphthalene fused to a benzene ring via a central five-membered-ring. The PAHs have been detected in shallow snow pits at Summit Station by multiple researchers (Jaffrezo *et al.*, 1994; Slater *et al.*, 2002; Von Schneidemesser *et al.*, 2008), so the presence of potential PAH chemical signals is consistent with known literature. There is similar overlap of Spectral Type L413_m and the Pony Lake fulvic acid sample, suggesting that materials deposited in the ice may be similarly complex. The Pony Lake fulvic sample is derived from polar microbial sources devoid of higher-order plants, whereas the Suwannee River fulvic acids are largely influenced by continental higher-order plants. Since Greenland is surrounded by continents with extensive boreal forests, it is likely that the Greenland ice sheet receives continental organic materials that may be preserved in firn and ice layers. Therefore, a continental higher-order organic end member sample, such as the Suwannee River fulvic acid, is a reasonable environmental comparative sample for this work.

Examples in Fig. 13 show that the Greenland ice sheet contains fluorescent signals over short and long wavelengths, which suggests that the materials deposited and preserved in ice vary in complex nature and may indicate diverse sources. Although similar spectral signals to laboratory standards or environmental mixtures do not confirm or quantify its presence, it suggests a similar chemical nature. In summary, all of our detected spectral features from the Summit borehole acquired by WATSON are similar to our measured laboratory microbial, chemical, and environmental material comparison samples that would be expected to be derived from microbes, pollution, aerosols, and eolian dusts delivered to the ice cap surface.

### 4.4. Estimates of instrument sensitivity

Using some speculative assumptions, we will try to estimate our instrument sensitivity based on literature concentrations of materials from Summit Station ice samples. Ice cores from the Greenland Ice Sheet coring project (GISP2) borehole at Summit Station provided a depth transect from the surface down to the base of the Greenland Ice Sheet ice sheet a little more than 3 km below the surface. For microbial concentrations, analysis of the GISP2 ice cores demonstrated that the cell counts varied from 10^4^ to 10^5^ cells/cm^3^ from the surface to a depth of 1.5 km below the surface, to 10^4^–10^6^ cells/cm^3^ from 1.5 to 2.5 km below the surface, and finally in the silt-infused bottom ice near the 3 km-deep base of the ice sheet, the cell counts went from 10^6^ to 10^9^ cells/cm^3^ (Miteva *et al.*, 2009). In the near-surface ice, previous work by Yung *et al.* (2007) with the GISP2 ice core found 7 × 10^3^ microbial cells/cm^3^ with nearly 400 endospores/cm^3^ at a depth of 93.96–94.21 m, which corresponded to an age of 295 years. Our field site was located only 7 km distant from the GISP2 location on the flat surface of the top of the ice sheet but several years later, these depths roughly correspond within 10 meters to our targeted borehole column.

We can use the microbial concentrations from the literature to speculatively estimate our instrument response based on our measured signal (Bhartia *et al.*, 2010). Our Map 1 was taken at a depth of 93.8 m: thus, from previous analyses (Yung *et al.*, 2007; Miteva *et al.*, 2009) we should expect roughly 10^4^ cells cm^3^ to be present. If we take the Map 1 spatial dimensions to be roughly 1 × 4 cm and assume an optical penetration depth of 1 cm (this value is conservatively decreased from the Eshelman *et al.*, 2019 estimate of 2 cm), then the entire ice volume scanned in Map 1 is 1 × 4 × 1 cm = 4 cm^3^. If Map 1 is representative of literature cell concentrations (Yung *et al.*, 2007; Miteva *et al.*, 2009), we would thus expect 4 × 10^4^ cells to be present in the Map 1 scanned volume. For the purposes of our estimate, we postulate that Spectral Types L325_t, L325_s, and L341_s are all microbial in nature, and that quantum yields are roughly similar. We, thus, estimate that the integrated λ_max_ signal intensity in Map 1 for these combined Spectral Types of 341,085 instrument counts corresponds to 4 × 10^4^ cells. This provides an estimated correspondence of ∼8.5 counts per cell. Assuming a LOD for these wavelengths of 300 instrument counts, this means it might be possible to detect a minimum of 35 cells in our sample if they are all in the same pixel of microbial confirmed signals. This estimated value is within the same order of magnitude to the value presented in the work of Eshelman *et al.* (2019) based on laboratory measurements of *Escherichia coli* in ice. At an estimated 8.5 counts per cell, this implies that Feature 2 in Map 1, with 206,492 integrated counts, would correspond to roughly 24,000 cells spread over an interrogation volume of 0.004 cm^3^ (0.004 cm^2^ spatial area × 1 cm estimated optical depth), which is a local concentration of 5.5 × 10^6^ cells cm^3^—more than a 500 × increase compared with the estimated bulk concentration.

We can use the same logic to speculate on the instrument response to longer wavelength signals. We speculate that the longer wavelength signals are due to PAHs. The PAHs have been detected in shallow trenches at Summit Station by multiple research groups (Jaffrezo *et al.*, 1994; Slater *et al.*, 2002; Von Schneidemesser *et al.*, 2008). The detected concentrations of PAHs in snow are low—on the order of a few nanograms per kilogram of bulk snow; past measurements required processing of large amounts of snow and highly sensitive detection techniques in the laboratory (Von Schneidemesser *et al.*, 2008). Of the PAHs detected, roughly 30% was fluoranthene, 20–30% was phenanthrene, 20–30% was pyrene, and 15–20% was naphthalene, with benzo[*a*]pyrene, benzo[*e*]pyrene, and benzo[*ghi*]perylene making up about 5–10% each (Jaffrezo *et al.*, 1994; Slater *et al.*, 2002) (for molecular structures of the base ring systems, see Fig. 14). It was noted that benzo[*a*]pyrene decomposes quickly in ice as evidenced by lower amounts detected from lower depths of a shallow snow trench, whereas fluoranthene and pyrene undergo moderate rates of decomposition in ice (Jaffrezo *et al.*, 1994). Phenanthrene is stable and would be expected to dominate after degradation of the other products. (The degradation pathways and fluorescence characteristics of the degradation products were not determined.).

Even though it is known to degrade moderately, we will speculate that the L374_sharp signal is from pyrene due to its spectral similarity (although there are differences between the method of collecting both spectra that preclude a positive identification). We will assume that pyrene is present in the Map 1 data at a concentration of 4 ng/kg snow = ca. 8 ng/kg ice [this is the highest concentration reported in the work of Slater *et al.* (2002), as a conservative figure we are assuming a 2 × compaction to glacial ice]. As stated earlier, assuming a conservative optical penetration depth of 1 cm, the entire ice volume scanned in Map1 is 1 × 4 × 1 cm = 4 cm^3^. Since our measured density was 0.84 g/cm^3^, we would expect 7 pg/cm^3^ of pyrene in our ice sample. For the volume in Map 1, this would be a total of roughly 28 pg present in Map 1's interrogated volume. We will use Spectral Type L374_sharp feature (ROI 13 in Fig. 8 and Table 4) as the sole indicator of pyrene in that volume to estimate the levels of detection. In Map 1, ROI 13 was the sole representative of Spectral Type L374_sharp and had a combined signal of 17,244 instrument counts at the λ_max_. This means that 616 counts of Spectral Type L374_sharp are the equivalent of 1 pg of pyrene. Assuming an LOD of 200 counts, our equivalent LOD is thus 0.33 pg of PAH in one pixel of the entire map scanned area. This is approximately equivalent to the highest reported sensitivity for a laboratory method (ca. 0.1 ng/kg^1^ snow) (Von Schneidemesser *et al.*, 2008).

These speculative estimates suggest that our *in situ* instrument has the potential to be highly sensitive and is able to detect both microbial and chemical signals at low native concentrations in a field setting at finer scales than can be measured by conventional ice core extraction and laboratory melting.

### 4.5. Prediction of hotspot analysis versus bulk dilution analysis

**FIG. 15.**
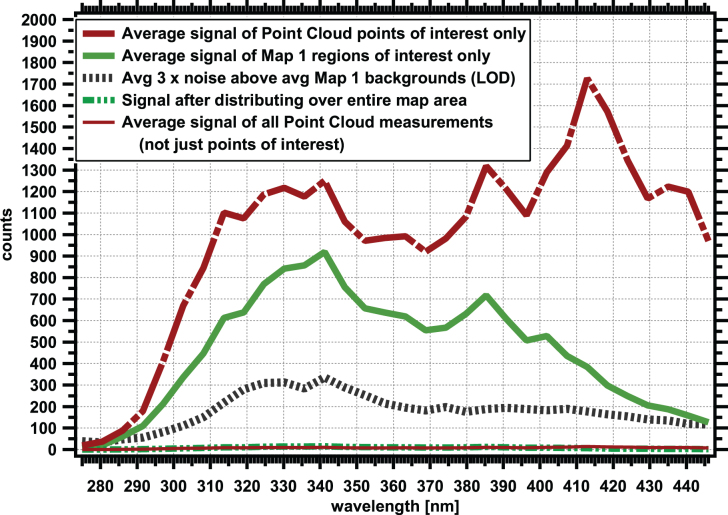
Plots showing average spectra. Dash-dotted red line shows the averaged spectra by combining all the point cloud points of interest and dividing by the number of point cloud hot spot pixels. Green line shows average spectra by combining all the map feature pixels together and dividing by the number of map feature pixels. The lower thin red line (just above zero) shows the integrated point cloud feature pixels divided by all the interrogated points in the point cloud. The lower green dot-dash line shows the average spectra across all the pixels in the map, including those without features. The shaded dash black line shows the average LOD (3 × noise) of the entire map area—it is roughly equivalent to a typical point cloud LOD. After full dilution, the diluted spectral signal is well below the LOD level and would not be detected. LOD, limit of detection. Color images are available online.

A future deep drilling mission through the icy crust of an Ocean World may sample melted ice during its journey on the way to the ocean, or it could examine hotspots embedded in he borehole walls: Which is better? This is the same question as comparing traditional bulk melt analysis with our fine resolution scanning. During traditional melt analysis, any contained fluorescent microbial or chemical hotspots are diluted into the volume melted and subsequently analyzed. We can examine the effect of dilution by using our collected data from the point clouds and Map 1 to compare detection levels between typical hotspots in the point cloud and map and a scenario if the entire datasets (point clouds and Map 1, separately) had been subjected to bulk melt analysis. To do this, we integrated the signals for all the point cloud and map points of interest and ROIs, respectively, and averaged them across all the hotspot pixels in each dataset to create an “average” hotspot spectrum. The averaged spectra per pixel for all the points of interest from the point cloud dataset are shown as a dot-dash brown line in Fig. 15, whereas the averaged ROIs spectrum per pixel for Map 1 data is the green line. For the averaged point cloud data, the spectrum has more of a response compared with Map 1 averaged spectrum at wavelengths above 400 nm; this is due to points of interest from Spectral Type 413_m and Spectral Type L418_d. These two Spectral Types are not observed in Map 1 data. Next, we took the summed signal intensity for the points of interest and ROIs and diluted them across all the point cloud points (127,799 pixels) and the Map 1 points (43,365 pixels), respectively. This represents the scenario where the integrated hotspots have been diluted by traditional bulk analysis. This exercise resulted in a signal dilution or decreased signal of 123 × less and 59 × less, for the point cloud and Map 1 data, respectively. The diluted spectra are shown as the double dot-dashed thin green line and the thin red line in Fig. 15 for the diluted point cloud data and the diluted Map 1 data, respectively. At this scale, the two lines are close to baseline and lie nearly on top of each other. A slight increase in signal intensity at wavelengths above 400 nm for the diluted point cloud data can barely be discerned.

In the same figure, we also plot the average LOD across the map area based on the average extracted backgrounds. We took all the local background spectra used for feature background subtraction and used the average standard deviation of the background signals. These were multiplied by a factor of 3 to generate the LOD (shown as a hashed black line in the figure). Any spectra falling below this limit would be not considered significant. Since all the diluted signals from either the point cloud or map pixels fall under this limit, they would not be detected.

This has important implications for future instrumentation and demonstrates the utility of detecting hotspots at fine scales. By examining hotspots concentrated in borehole ice rather than bulk melt dilution, we can gain 50–100 × in signal intensity. Depending on the instrument threshold, this could be the difference between detecting and not detecting a signal. *In situ* ice scanning also maintains spatial integrity and allows more detailed analysis on associations, layering (or lack thereof), and spot distribution. This analysis, thus, provides impetus for future *in situ* scanning and spatial mapping of ice boreholes compared with traditional bulk analysis.

## 5. Conclusions

Our DUV fluorescence mapping spectrometer revealed diverse spectral features from 0 to 107 m in both firn and glacial ice. We used both a linear point cloud scanning mode and a raster mapping mode with a coupled drill-instrument combination. Both instrument modes detected spectrally diverse features consistent with individual, stochastically distributed <20 mm globules of material. Overall, the frequency of the hotspot intensities followed a power law, with intense signals only a few percent of the detected signals. From point cloud analysis, we found that most of the features were small with a few larger features measuring up to 60 mm in dimension. We did not see any of these larger features in our raster maps, instead noting that most features were <2 mm in size. We found Spectral Type-dependent differences in hit rate frequencies as well as differences in frequencies above and below the firn-glacial ice transition. The Spectral Types we identified had spectra that are similar to microbes, lignin-phenols, fused-ring aromatic molecules, including PAHs, and environmentally complex fulvic acids of microbial and multicellular origin.

We found no evidence of fluorescent material emplaced in layers, at least at the fine scales examined. There was no apparent correlation between the size of the feature and its depth. Both map and point cloud data had features of roughly the same distribution in shallow firn and in deeper glacial ice. This suggests that the mechanisms of emplacement favor smaller features and may occur at shallow depth, and then “lock in” early in the process. The distribution of most spectral types appears the same in glacial as for firn ice, although some exceptions were noted.

We successfully demonstrated *in situ* detection and spectral analysis of diverse punctate chemical signals similar to microbial and other organic molecules in a drilled ice borehole at Summit Station, Greenland. Data obtained from Summit Station show that our DUV fluorescence mapping spectrometer with 100 μm spacing can spatially resolve spectrally unique features, whereas traditional bulk melt analysis removes spatial context and potentially dilutes the signal below instrumentation limits of detection. Our DUV-based technique can be useful for localizing the emplacement of organic material in icy environments on Earth. This technique could also be key to the detection of astrobiologically relevant organic molecules for a future mission to the icy crusts of the Ocean Worlds such as Europa, Enceladus, and Titan, or perhaps the polar caps of Mars.

## Supplementary Material

Supplemental data

Supplemental data

Supplemental data

Supplemental data

## Abbreviations Used

λ_max_: lambda max
DUV: deep-ultraviolet
EEMs: excitation–emission matrices
GISP2: Greenland Ice Sheet Project 2
LOD: limit of detection
PAH: polycyclic aromatic hydrocarbon
PMT: photomultiplier tube
ROIs: regions of interest
WAIS: West Antarctic Ice Sheet
WATSON: Wireline Analysis Tool for the Subsurface Observation of Northern ice sheets

## Notes

Associate Editor: Victor Parro
