# Supplementary material for: Subsurface In Situ Detection of Microbes and Diverse Organic Matter Hotspots in the Greenland Ice Sheet
Source: Astrobiology. 2020 Oct 9;20(10):1185–211. doi: 10.1089/ast.2020.2241 (PMC7591382; doi:10.1089/ast.2020.2241)
Supplement: Supplemental data [file Supp_Data.zip › Supp_Data.pdf]

Supplementary Data

Supplementary Data S1

Point Cloud Dataset

(Refer to Excel Table below: Malaska_WATSON_ Supplement_S1_Total_corrected_pointcloud_dataset.csv)

The data were background-corrected, as described in Section 2 in the main text. The comma-separated text file contains the following fields: Point_Cloud_Map, Point_ Cloud_name, row_num, depth_m, rot_from_magN_deg, timestamp, Band_1_bckgnd_corr….Band_32_bckgnd_corr, Band_1_max_std_dev….Band_32_max_std_dev. Each row records the background-corrected data returned from a laser shot. ''Point_Cloud_Map'' numbering refers to Table 1 in the main text, ''Point_Cloud_name'' is a more detailed Point Cloud identifier and has the syntax ''Pointcloud_''+ date of acquisition [yyyy_mm_dd]+time of acquisition [hh_mm_ss]—dates and times were local time, ''row_num'' is a row counter that alternates, ''depth_m'' is the depth from the surface as measured by the drill encoder, and ''rot_from_magN_deg'' is the degree of rotation of the optical view clockwise from magnetic N recorded as part of drill system telemetry. "Timestamp" is the time of acquisition of that particular measurement. The fields "Bands_1_bckgnd_ corr''….''Bands_32_bckgnd_corr'' record the background-corrected responses in Bands 1 through Band 32. The fields "Band_1_max_std_dev''….''Band_32_max_std_dev'' record the maximum standard deviation from the regions selected for determining the background for adjustment.

The band correspondence is shown in Supplementary Table S1, which lists the band number and the wavelength center in nm.

Supplementary Data S2

Extracted Points of Interest from the Point Cloud Dataset

(Refer to Excel Table below: Malaska_WATSON_ Supplement_S2_Points_of_interest.csv)

Supplementary Data S2 is the dataset of points of interest extracted from the entire point cloud data (Supplementary Data S1) set such that these points that have three consecutive points above 10 × the maximum standard deviation of the background regions are used to develop the background curve. This table is a comma-delimited text file (.csv) with the following fields: ''Point_Cloud_Map'' (refer to Map number in Table 1 of the main text), ''Point_Cloud'' (syntax is ''Pointcloud_''+date acquired[yyyy_mm_dd] + time acquired [hh_mm_ss]), ''row_num'' (ref to S1), ''depth_M'' (level of optical path below the surface in m), ''rot_ from_magN_deg'' (rotational angle in deg offset from Mag N), ''Major_Spectral_Type'' (dominant Spectral Type, refer to Table 2 in the main text), and ''minor_Spectral_Type'' (minor Spectral Type component, if present, refer to Table 2 in the main text). The ''lambda_max_band'' identifies the band number used for the lambda max (λ_{max}) value extraction (note that this may not be the true λ_{max} for that particular spectrum), whereas ''lambdamax_strength'' indicates the corrected corresponding value extracted from the band data fields. The fields ''Band_1_bckgnd_corr''…. Bands_32_bckgnd_corr fields record the background-corrected responses in Bands 1 through Band 32. The fields ''Band_1_max_std_dev''….''Band_32_max_std_dev'' record the maximum standard deviation from the regions selected for determining the background for adjustment.

The Band numbers to wavelength correspondence is the same as listed in Supplementary Table S1.

Supplementary Data S3

Frequency Distribution of Signal Intensities in Point Cloud and Map Data

We determined the frequency distribution of selected points in the point cloud and map according to strength of the signal at the lambda maxima. This is shown in Supplementary Fig. S2. The signal intensities spanned over four orders of magnitude. The signal intensity at the lambda max (λ_{max}) was placed in bins of 100 from 0 to 3000, bins of 1000 from 3000 to 10,000, and bins of 10,000 from 10,000 to 70,000. In the plot, the percentages of all identified signals above a given instrument count value (x axis) are plotted. From the 1039 points of interest in the point cloud dataset, only 3 points had signals >40,000 counts. Weak signals (<2000 instrument counts) predominate over stronger ones. For example, 70% of all points of interest in the point cloud dataset are within 3000 instrument counts of the background, and 95% of all points are below 20,000 background-subtracted instrument counts. The curve for the points of interest is only adjusted slightly (dotted line in plot) when the potential 85 m impurity (Spectral Type 385_d_complex) is removed. Due to the uniformity of signal intensity (most signals of the Spectral Type had signal intensities from 708 to 5390, with an average value of 1892 and a standard deviation of 389) of this impurity, the slight skew due to these signals can be noted in the zoom region of Supplementary Fig. S1. Of the most intense signals, several Spectral Types with a variety of lambda maxes were noted—they did not come from any one spectral class. For the Map 1 pixels extracted from the regions of interest (ROIs), only 3% of the hotspot pixels had signals more than 5000 counts; these pixels all were in ROI 2 and were of Spectral Type 325_t (see also Fig. 10A in the main text). The bulk (>80%) of the pixels from the Map 1 ROI had signal intensities less than 1000 counts. Comparison of the intensity distribution of the Map 1 data to that from the point cloud data shows that the Map 1 pixels have a lower intensity distribution than those found in the point cloud data. Both sets showed the same trend of many weak signals, and a few strong signals.

The distribution signal intensities at the λ_{max} are plotted for both Point Cloud (blue and dashed black line) and Map 1 data (red line) and are shown in Supplementary Fig. S2. The

plot shows the percentage of points of interest (or pixels in ROIs for the Map 1 data) that are above a certain instrument signal intensity. As the intensity level increases, fewer pixels are above that intensity. The distribution follows a rough power law, with many low signals and a few high signals. We also show a distribution curve for point cloud data, where the points of Spectral Type L385_d complex have been removed. All the curves are roughly parallel.

Supplementary Data S4

Map 1 Data

(Refer to Excel Table below: MALASKA_WATSON_Supplement_S4_Map1_bias_corrected_full_map_pixels.csv)

Map 1 data were collected as described in Section 2 in the main text. Dark-subtracted but non-background corrected values are reported here. The comma-separated text file contains the following fields: entry, x_coord_pixel, y_coord_pixel, x_coord_mm, y_coord_mm, depth_m, rot_from_magN_deg, Band_1….Band_32. ''Entry'' is a row counter for each of the rows—for convenience, the pixels are listed from top to bottom, reading left to right along each row. The fields ''x_coord_pixel'' and ''y_coord_pixel'' count the number of pixels in *x* and *y* direction, respectively, from an origin at the lower left of the raster map. The fields ''x_coord_mm'' and ''y_coord_mm'' provide distance in mm from an origin at the lower left of the raster map (each pixel is 0.1 mm across). ''depth_m'' is calculated from the drill start position and is subtracted (thus upwards, less deep) from the origin. ''rot_from_magN_deg'' is the calculated degrees of rotation of the optical view clockwise from magnetic N, with the start position recorded as part of drill system telemetry for the lower right corner of the map, and the angle calculated from this position based on map distance along the curved borehole wall of diameter 11.18 cm. The fields ''Band_1''….''Bands_32'' fields record the raw signal responses in Bands 1 through Band 32. The Band numbers to wavelength correspondence are the same as listed in Supplementary Table S1.

Supplementary Data S5

Region of Interest Pixel Data Table for Map 1

(Refer to Excel Table below: MALASKA_WATSON_Supplement_S5_Map1_region_of_interest_pixels.csv)

The Map 1 data (Supplementary Data S4) was background-corrected, as described in Section 2 in the main text. The comma-separated text file contains the following fields: entry_no, Region_ID, Region_pixel,, x_coord_pixel, y_coord_pixel, x_coord_mm, y_coord_mm, Spectral_Type, lambda_max_band, lambda_max_strength, Band_1_bckgnd_corr….Band_32_bckgnd_corr, Band_1_max_std_dev….Band_32_max_std_dev. Each row records the background-corrected data returned from a laser shot during the raster scan, and the regions of interest are listed from top to bottom of Map 1. The field ''entry_no'' is a counter for all of the entries. ''Region_ID'' refers to the region in Table 4 and Fig. 9 in the main text. ''Region_pixel'' is an internal counter for pixels that make up each region. ''x_coord_pixel'' and ''y_coord_pixel'' counts the number of pixels in *x* and *y* direction, respectively, from an origin at the lower left of the raster map. ''x_coord_mm'' and ''y_coord_mm'' provide distance in mm from an origin at the lower left of the raster map (each pixel is 0.1 mm across). The field ''Major_Spectral_Type'' records the dominant Spectral Type (refer to Table 3 in the main text). The ''lambda_max_band'' identifies the band number used for the λ_{max} value extraction (note that this may not be the true λ_{max} for that particular spectrum), whereas ''lambdamax_strength'' indicates the corrected corresponding value extracted from the band data fields. The fields ''Band_1_bckgnd_corr''….Bands_32_bckgnd_corr fields record the background-corrected responses in Bands 1 through Band 32. The fields ''Band_1_max_std_dev''….''Band_32_max_std_dev'' record the maximum standard deviation from the regions selected for determining the background for adjustment. The Band numbers to wavelength correspondence is the same as listed in Supplementary Table S1.
