# Supplementary material for: Subsurface In Situ Detection of Microbes and Diverse Organic Matter Hotspots in the Greenland Ice Sheet
Source: Astrobiology. 2020 Oct 9;20(10):1185–211. doi: 10.1089/ast.2020.2241 (PMC7591382; doi:10.1089/ast.2020.2241)
Supplement: Supplemental data [file Supp_Table2.pdf]

Supplementary Table S2. Multipoint Features from the Point Cloud Dataset

Entry No.	*Depth (m)*	*Rotational orientation (degrees from magnetic N)*	*Point Cloud ID (Table 1 in the main text)*	*No. of consecutive shots with significant signal*	*Post spacing (mm)*	*Maximum estimated size from spacing (mm)*	*Average signal intensity at lambda max (counts)*	*Spectral type (Table 3 in the main text)*
1	4.113	28	1	3	1403	5.6	5982	L385_m
2	4.665	38	1	2	1403	4.2	1375	L385_m
3	6.402	32	1	2	1403	4.2	7315	L385_m
4	6.410	32	1	2	1403	4.2	13,700	L325_t
5	7.262	32	1	3	1403	5.6	2391	L325_t
6	9.826	69	2	2	183	0.5	458	L413_m
7	9.831	69	2	2	183	0.5	543	L385_m
8	9.833	69	2	2	183	0.5	508	L385_m
9	9.841	69	2	2	183	0.5	713	L385_m
10	9.888	68	2	2	183	0.5	511	L413_m
11	9.925	68	2	2	183	0.5	1305	L413_m
12	9.926	68	2	9	183	1.8	1681	L413_m
13	9.935	68	2	3	183	0.7	622	L385_m
14	9.968	68	2	2	183	0.5	600	L413_m
15	9.970	68	2	2	183	0.5	518	L413_m
16	9.991	67	2	2	183	0.5	488	L413_m
17	10.010	67	2	2	183	0.5	597	L413_m
18	10.011	67	2	2	183	0.5	467	L413_m
19	10.018	67	2	2	183	0.5	413	L413_m
20	10.033	67	2	2	183	0.5	551	L413_m
21	10.219	64	2	2	183	0.5	1130	L341_a
22	10.307	63	2	4	183	0.9	855	L413_m
23	20.166	71	3	36	121	4.5	1368	L325_t
24	32.177	110	6	2	1536	4.6	907	L325_t
25	32.625	124	6	2	1536	4.6	1210	L314_d
26	33.948	158	6	2	1536	4.6	823	L374_m
27	34.523	153	6	3	1536	6.1	1368	L374_m
28	34.562	155	6	4	1536	7.7	923	L385_m
29	37.037	49	5	3	5321	21.3	2012	L314_s
30	37.086	49	5	4	5321	26.6	4736	L314_s
31	37.183	49	5	2	5321	16.0	1884	L314_s
32	37.289	50	5	7	5321	42.6	7483	L314_s
33	37.360	50	5	3	5321	21.3	2440	L325_t
34	37.480	50	5	2	5321	16.0	1103	L314_s
35	37.654	51	5	6	5321	37.2	11,584	L314_s
36	37.738	51	5	2	5321	16.0	2574	L314_s
37	37.811	51	5	2	5321	16.0	1228	L314_s
38	37.863	51	5	13	5321	74.5	4632	L314_s
39	38.109	51	5	3	5321	21.3	2903	L314_s
40	38.171	52	5	3	5321	21.3	1342	L314_s
41	38.431	52	5	9	5321	53.2	9746	L314_s
42	38.791	53	5	3	5321	21.3	1114	L314_s
43	39.145	54	5	3	5321	21.3	3789	L314_s
44	55.856	74	9	6	1544	10.8	851	L314_s
45	55.863	78	9	4	1544	7.7	1513	L314_s
46	64.090	69	8	2	5042	15.1	1272	L385_m
47	84.231	346	12	2	2441	7.3	15,428	L385_m
48	84.258	85	11	6	4813	33.7	5417	L385_m
49	84.582	34	14	6	125	0.9	731	L358_d
50	84.585	34	14	44	125	5.6	798	L358_d
51	84.597	345	12	3	2441	9.8	1130	L385_m
52	84.926	34	14	237	125	29.8	1870	L385_d_complex
53	85.038	346	12	2	2441	7.3	1177	L385_m
54	85.865	348	12	3	2441	9.8	813	L385_m
55	86.250	348	12	2	2441	7.3	7124	L385_m
56	89.028	72	15	2	1370	4.1	959	L325_t
57	89.032	72	15	2	1370	4.1	863	L325_t
58	89.572	6	16	3	1164	4.7	2065	L314_d

(*continued*)

Supplementary Table S2. (Continued)

Entry No.	*Depth (m)*	*Rotational orientation (degrees from magnetic N)*	*Point Cloud ID (Table 1 in the main text)*	*No. of consecutive shots with significant signal*	*Post spacing (mm)*	*Maximum estimated size from spacing (mm)*	*Average signal intensity at lambda max (counts)*	*Spectral type (Table 3 in the main text)*
59	91.557	10	16	3	1164	4.7	2051	L314_d
60	91.628	69	15	2	1370	4.1	1265	L385_m
61	91.776	69	15	2	1370	4.1	1018	L385_m
62	93.309	68	15	2	1370	4.1	1012	L385_m
63	93.700	48	17	3	153	0.6	594	L325_t
64	93.704	48	17	4	153	0.8	197	L325_t
65	93.705	68	15	3	1370	5.5	1214	L385_m
66	93.729	48	17	36	153	5.7	2939	L385_m
67	93.789	48	17	4	153	0.8	486	L385_m
68	93.815	48	17	2	153	0.5	115	L314_d
69	94.769	48	22	2	119	0.4	303	L385_m
70	94.770	48	22	4	119	0.6	412	L385_m
71	94.778	48	22	2	119	0.4	542	L385_m
72	98.050	46	23	60	159	9.7	974	L325_t
73	98.055	46	23	20	159	3.3	2159	L385_br_d
74	98.191	46	23	2	159	0.5	2744	L325_t
75	98.600	20	25	2	143	0.4	1009	L325_t
76	98.955	22	25	3	143	0.6	526	L314_s
77	101.117	96	27	43	782	34.4	21,207	L418_d
78	101.837	94	27	28	782	22.7	801	L341_s
79	105.235	47	30	3	157	0.6	414	L325_t
80	105.480	327	31	7	165	1.3	893	L413_m

Point cloud dataset is Supplementary Data S1.
