# Supplementary material for: Subsurface In Situ Detection of Microbes and Diverse Organic Matter Hotspots in the Greenland Ice Sheet
Source: Astrobiology. 2020 Oct 9;20(10):1185–211. doi: 10.1089/ast.2020.2241 (PMC7591382; doi:10.1089/ast.2020.2241)
Supplement: Supplemental data [file Supp_Figs1-2.pdf]

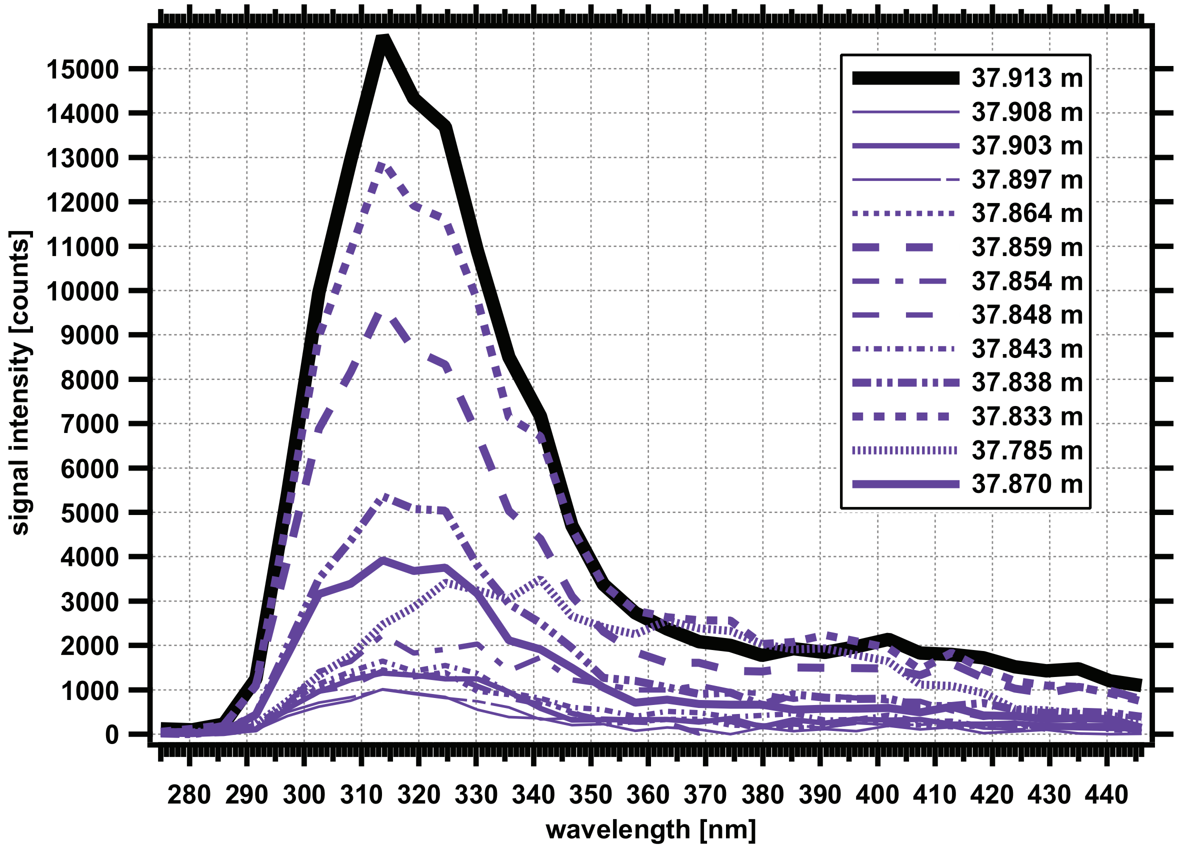

SUPPLEMENTARY FIG. S1. Fluorescence spectral data of points composing entry 38 (Supplementary Table S2). The spectra from these points are most similar to Spectral Type L314s (refer to Fig. 6 in the main text for comparison).

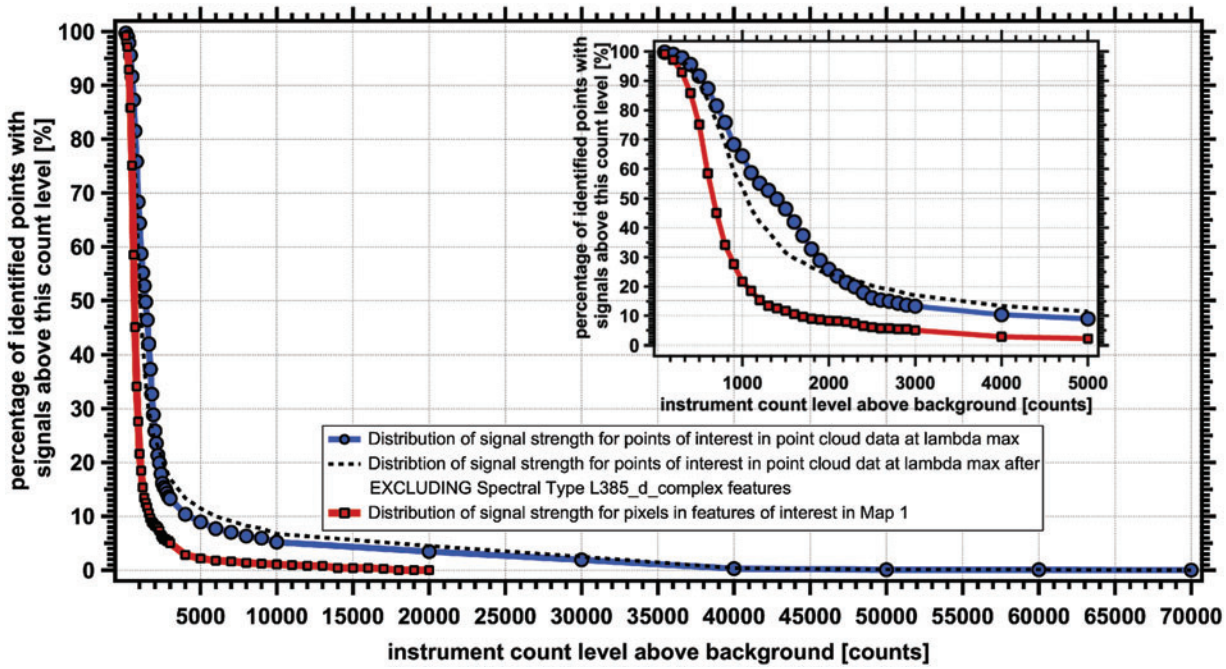

SUPPLEMENTARY FIG. S2. Distribution of signal at λ_{max} as percentage of total signal for point cloud data (blue line and markers and dashed black line) and individual pixels from regions of interest in the map data (red markers and line). The plot shows that there are many weak signals, but fewer strong signals. Inset shows detail for plots below 5000 instrument counts, which accounts for 80–90% of the observed signals. Blue markers connected by a blue line are for all identified points in the point cloud dataset. Black dashed line is for the same dataset after the exclusion of Spectral Type L385_d_complex that was found only at 84.9 m depth. λ_{max}, lambda max.
