# Supplementary material for: Subsurface In Situ Detection of Microbes and Diverse Organic Matter Hotspots in the Greenland Ice Sheet
Source: Astrobiology. 2020 Oct 9;20(10):1185–211. doi: 10.1089/ast.2020.2241 (PMC7591382; doi:10.1089/ast.2020.2241)
Supplement: Supplemental data [file Supp_Table1.pdf]

Supplementary Table S1. Band Correspondence for Wireline Analysis Tool for the Subsurface Observation of Northern Ice Sheets Instrument

WATSON Band No.	*Wavelength center (nm)*
1	275
2	280.5
3	286
4	291.6
5	297.1
6	302.6
7	308.1
8	313.7
9	319.1
10	324.6
11	330.2
12	335.7
13	341.2
14	346.7
15	352.2
16	357.7
17	363.3
18	368.8
19	374.3
20	379.8
21	385.3
22	390.8
23	396.3
24	401.9
25	407.4
26	412.9
27	418.4
28	423.9
29	429.4
30	435
31	440.5
32	446

WATSON = Wireline Analysis Tool for the Subsurface Observation of Northern ice sheets.
